# Novel Small Molecule Tyrosine Kinase 2 Pseudokinase Ligands Block Cytokine-Induced TYK2-Mediated Signaling Pathways

**DOI:** 10.3389/fimmu.2022.884399

**Published:** 2022-05-20

**Authors:** Yu Zhou, Xin Li, Ru Shen, Xiangzhu Wang, Fan Zhang, Suxing Liu, Di Li, Jian Liu, Puhui Li, Yinfa Yan, Ping Dong, Zhigao Zhang, Heping Wu, Linghang Zhuang, Rasheduzzaman Chowdhury, Matthew Miller, Mena Issa, Yuchang Mao, Hongli Chen, Jun Feng, Jing Li, Chang Bai, Feng He, Weikang Tao

**Affiliations:** ^1^ R & D Center, Eternity Bioscience Inc., Cranbury, NJ, United States; ^2^ R & D Center, Shanghai Hengrui Pharmaceutical Co. Ltd., Shanghai, China

**Keywords:** Tyrosine Kinase 2 (TYK2), Janus Kinases (JAK), JAK homology 2 (JH2) pseudokinase regulatory domain, cytokine pathways, small molecule inhibitors, selectivity

## Abstract

A member of the Janus kinase (JAK) family, Tyrosine Kinase 2 (TYK2), is crucial in mediating various cytokine-signaling pathways such as interleukin-23 (IL23), interleukin-12 (IL12) and type I Interferons (IFN) which contribute to autoimmune disorders (e.g., psoriasis, lupus, and inflammatory bowel disease). Thus, TYK2 represents an attractive target to develop small-molecule therapeutics for the treatment of cytokine-driven inflammatory diseases. Selective inhibition of TYK2 over other JAK isoforms is critical to achieve a favorable therapeutic index in the development of TYK2 inhibitors. However, designing small molecule inhibitors to target the adenosine triphosphate (ATP) binding site of TYK2 kinase has been challenging due to the substantial structural homology of the JAK family catalytic domains. Here, we employed an approach to target the JAK homology 2 (JH2) pseudokinase regulatory domain of the TYK2 protein. We developed a series of small-molecule TYK2 pseudokinase ligands, which suppress the TYK2 catalytic activity through allosteric regulation. The TYK2 pseudokinase-binding small molecules in this study simultaneously achieve high affinity-binding for the TYK2 JH2 domain while also affording significantly reduced affinity for the TYK2 JAK homology 1 (JH1) kinase domain. These TYK2 JH2 selective molecules, although possessing little effect on suppressing the catalytic activity of the isolated TYK2 JH1 catalytic domain in the kinase assays, can still significantly block the TYK2-mediated receptor-stimulated pathways by binding to the TYK2 JH2 domain and allosterically regulating the TYK2 JH1 kinase. These compounds are potent towards human T-cell lines and primary immune cells as well as in human whole-blood specimens. Moreover, TYK2 JH2-binding ligands exhibit remarkable selectivity of TYK2 over JAK isoforms not only biochemically but also in a panel of receptor-stimulated JAK1/JAK2/JAK3-driven cellular functional assays. In addition, the TYK2 JH2-targeting ligands also demonstrate high selectivity in a multi-kinase screening panel. The data in the current study underscores that the TYK2 JH2 pseudokinase is a promising therapeutic target for achieving a high degree of biological selectivity. Meanwhile, targeting the JH2 domain represents an appealing strategy for the development of clinically well-tolerated TYK2 inhibitors that would have superior efficacy and a favorable safety profile compared to the existing Janus kinase inhibitors against autoimmune diseases.

## Introduction

Cytokine signaling transduction plays a pivotal role in controlling the growth, differentiation, function, and communication of immune cells (1–4). Multiple cytokine-signaling pathways are tightly regulated by the actions of receptor-bound JAKs and the signal transducers and activators of transcription (STATs) (5–7). Dysfunctional cytokine-JAK/STAT activities have been demonstrated as hallmarks of numerous autoimmune disorders and inflammatory diseases (6–10). In the past decade, small molecule JAK inhibitors have been shown to be crucial therapeutic agents in the treatment of autoimmune diseases. Recent advances in the development of JAK inhibitors have provided significant benefits to some patients with autoimmune and chronic inflammatory diseases (11–14). Nevertheless, many existing JAK inhibitors representing the current standard of care have safety concerns that limit their chronic use. The safety risks of JAK inhibitors can be also associated with a considerable decline in host defense or aggravating lipid metabolism disorders, which can result in an increased risk of serious infections or blood clots and even life-threatening cardiovascular (CV) disease (15–20). Given the shortcomings of current therapeutics, the unmet medical need remains high for many patients suffering from autoimmune disorders. Compelling evidence demonstrates that the relationship between JAK isoform selectivity and suppression of distinct cytokine responses (**Supplementary Figure 1**) could provide a mechanistic basis for the improvement of efficacy and safety profile of JAK inhibitors (15, 21). JAK inhibitors with preferable selectivity are of particular interest to achieve better efficacy and an improved safety profile. However, the traditional design of selective ATP site JAK inhibitors has encountered challenges owing to the high homology among the kinase domains of the JAK family proteins (22–27).

TYK2, the first identified member of the JAK family, is a major participant in regulating various signal transduction pathways downstream of the cytokine receptors for IL23, IL12, and type I IFNs (2, 4, 24, 28–32). IL23 has been widely recognized as a major effector protein in determining the survival and expansion of pathogenic T helper 17 (Th17) cells, triggering the secretion of pro-inflammatory cytokines and thus serving as a key driver in the subsequent inflammatory cascade during immune disorders (3, 8, 30, 32). IL12 is critical for T helper 1 (Th1) development and drives the secretion of IFNγ, an essential molecule participating in systemic autoimmune disorders such as SLE and lupus nephritis (4). Type I IFNs have been demonstrated to induce monocytes and antigen-presenting dendritic cell differentiation, which is believed to be an important cellular mechanism to drive the function of autoreactive B and T cells in lupus and other autoimmune disorders (28). As a critical intracellular component of the IL12, IL23 and type I IFN cytokine cascades, TYK2 plays pivotal roles in autoimmune disorders including psoriasis (Ps), inflammatory bowel disease (IBD), systemic lupus erythematosus (SLE), lupus nephritis, multiple sclerosis (MS), etc. (2–4, 8, 22, 25). Indeed, emerging research evidence demonstrated the importance of TYK2 in the pathogenesis of the aforementioned autoimmune diseases (25, 28, 29, 33–37). For example, TYK2-deficient or chemically-inhibited rodents were revealed to be resistant to experimental autoimmune disease models such as MS and Rheumatoid arthritis (RA) (28, 38, 40). In human studies, catalytically impaired TYK2 variants (rs12720356, and rs34536443) were found to be protective against childhood and adult onset of SLE in the Mexican Mestizo population (37). TYK2 SNP mutations were also identified to be associated with SLE in individuals of Nordic, UK, and Han Chinese ancestry (36), as well as MS patient susceptibility (41–43). Furthermore, despite the conflicting evidence regarding the phenotypic impact of genetic variants in the TYK2 gene on immunodeficiency (44–48), the study by Dendrou et al. revealed that TYK2 rs34536443 variant homozygosity shows no association with increased hospitalization due to mycobacterial, bacterial, viral or fungal infection (34). The performed genetic meta-analysis, molecular, cellular, *in vivo* and structural functional, as well as epidemiological studies suggest that rs34536443 homozygosity could drive an optimal degree of TYK2 immune signaling. The remained TYK2 signal is low enough to allow protection against autoimmunity but still sufficient to prevent detrimental immunodeficiency (34). Similarly, the genome-wide association studies (GWAS) have not observed increased risk of pathogen infection-associated hospitalization in human patients that carry the TYK2 variants which are resistant to inflammatory diseases (49). Moreover, Fuchs et al. found that preserved type-III IFN responses and Natural Killer (NK)-cell function may contribute to antiviral protection even with TYK2 deficiency and lead to a mild human phenotype (50). Taken together, the research and genetic evidence supports the merit of blocking TYK2 as a promising therapeutic strategy against autoimmune-disorders with optimal balance between efficacy and safety.

**Figure 1:**
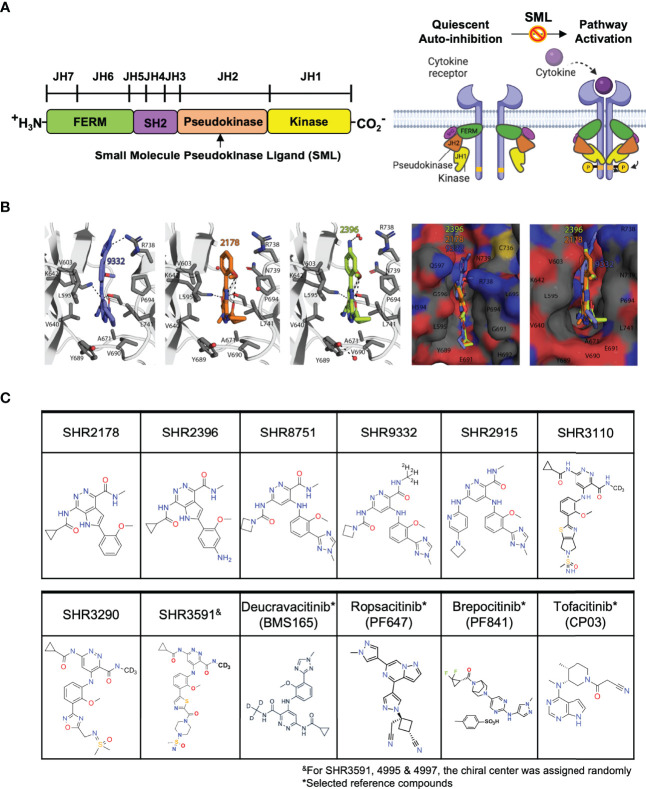
Small molecule ligands bind to the TYK2 JH2 pseudokinase domain. **(A)** Domain structural illustration of TYK2 and schematic model of cytokine-mediated receptor/TYK2 activation: TYK2 protein contains 4 components: the kinase domain (JH1), the pseudokinase domain (JH2), the FERM (Four-point-one protein, Ezrin, Radixin, Moesin) domain, and the Src homology (SH2) domain; In the quiescent state, TYK2 kinase domain (JH1) activity is autoinhibited by its pseudokinase JH2 domain through intermolecular regulation. Upon cytokine (e.g., IL12, IL23, IFNα) stimulation, TYK2/STAT pathway is initiated by extracellular binding of cytokines with their cognate receptors, which results in apposition of receptor-associated TYK2 and triggering their enzymatic activity. Activated TYK2 further mediates phosphorylation of STATs on their tyrosine residues, leading to the cascade of down-stream cytokine signaling, including STAT dimerization, nuclear translocation, DNA binding and target gene induction. Small molecule TYK2 JH2 ligands block TYK2-associated cytokine signaling by binding to the TYK2 pseudokinase domain and allosterically inhibiting its kinase activities. This prevents TYK2 from phosphorylating STATs and other substrates, so that cytokine-induced intracellular signals cannot be transduced; **(B)** Model of the TYK2 pseudokinase domain bound to TYK2 ligands, SHR2396, 2178 and 9332. TYK2 pseudokinase domain residues corresponding to those of protein kinases normally involved in catalytic machinery are shown in stick; **(C)** Chemical structure of small molecule TYK2 JH2 ligands and JAK kinase inhibitors described in this study [SHR2178, 2396, 8751, 9332, 2915, 3290, 3110, 3591, Deucravacitinib (BMS986165, TYK2 pseudokinase ligand), Ropsacitinib (PF647, TYK2-IN-8, TYK2 kinase inhibitor), Brepocitinib (PF841, TYK2/JAK1 inhibitor) and Tofacitinib (JAK1/JAK3 inhibitor)].

The structure of TYK2 contains a complex architecture with multiple domains participating in both inter- and intramolecular interactions which transit cytokine receptor-mediated activation to its catalytic domain (**Figure 1A**) (27, 51, 52). TYK2 contains four distinct domains: (i) the N-terminal Ezrin, Radixin, Moesin (FERM) and (ii) Src homology 2 (SH2) domains (which constitute the receptor-binding module) followed by C-terminal (iii) JH2 pseudokinase and (iv) JH1 kinase domains (**Figure 1A**) (51, 52). The JH2 pseudokinase domain has a canonical kinase fold but lacks catalytic activity. Rather than acting catalytically, the JH2 domain plays a pivotal role in regulating the receptor-mediated activation of the adjacent JH1 kinase domain through intermolecular autoinhibition. Evidence suggests that stabilizing the TYK2 JH2 pseudokinase domain led to a protein conformational change that prevents receptor-mediated activation and hinders activity of the TYK2 JH1 catalytic domain by blocking relief of intermolecular autoinhibitory interactions between the TYK2 JH2 pseudokinase and JH1 kinase domains (**Figure 1A**) (51, 52). In the JAK family, it was reported that the pseudokinase and kinase domains in JAK2 coimmunoprecipitate and lead to co-expression of the JAK2 JH2 pseudokinase domain suppresses activity of the isolated JH1 kinase domain (53). Additionally, deletion of the pseudokinase domain in JAK2 and JAK3 elevates basal kinase activity and increases signaling through cognate receptors (53–55).

The TYK2 inhibitor, Deucravacitinib (BMS986165), is currently the compound most advanced clinically, which selectively binds to the JH2 regulatory domain of TYK2 and suppresses TYK2 associated cytokine pathways (22, 27, 52, 56–59). Deucravacitinib was demonstrated to be highly efficacious in phase 2 and 3 psoriasis trials with good safety profiles (58). In this study, we employ an approach that deviates from the typical ATP binding site-targeting method and instead we develop a series of small-molecule TYK2 ligands that selectively target the JH2 pseudokinase regulatory domain of TYK2 (**Figure 1**). The TYK2 JH2 pseudokinase ligands inhibit the TYK2 JH1 catalytic domain activity through the intermolecular JH2-JH1 interaction. Our investigations demonstrated that the compounds are highly selective for the TYK2 pseudokinase domain and are able to effectively block the TYK2-mediated receptor-stimulated pathway activities in immune cells. This is achieved with a minimal impact on the JAK-related cellular cytokine-functional pathways. The mechanistic approach adopted for the TYK2 JH2 ligands is distinct from traditionally designed JAK and TYK2 inhibitors which target the ATP binding site in the JH1 domain. The new series of TYK2 JH2 ligands specifically bind to the JH2 pseudokinase regulatory domain of TYK2, thereby maintaining TYK2 in an inactive conformation, leading to inhibition of its kinase activity.

## Materials and Methods

### KdELECT Competition Binding Assays

The KdELECT competition assays were performed to test the binding affinity of compounds to the JH1 kinase region or JH2 pseudokinase region in TYK2 or JAK by ligand competition with JH1 or JH2 protein segments. The assay was carried out at Eurofins DiscoverX Corporation following the established standard protocol (DiscoverX, San Diego, CA). In this assay, compounds that bind to the kinase or pseudokinase protein regions prevent the protein binding to the immobilized ligand and reduce the amount of kinase matter captured on the solid surface. Conversely, compounds that do not bind to the kinase have no effect on the amount of kinase captured on the solid support. Dissociation constants (Kds) for test compound-kinase interactions are calculated by measuring the amount of kinase captured on the solid support as a function of the test compound concentration. Briefly, the JH1 or JH2 protein segments were either expressed on T7 phage coat and tagged with a quantitative polymerase chain reaction (qPCR) detection amplicon by imbedding sequence in phage genome, or produced in Human Embryonic Kidney 293 (HEK 293) cells as fusion with nuclear factor kappa light chain enhancer of activated B cells (NFkB) DNA binding domain and tagged with qPCR detection amplicon through interaction with NFkB. Streptavidin-coated magnetic beads were treated with biotinylated small-molecule ligands for 30 minutes at room temperature to generate affinity resins for the protein-kinase binding assays. Streptavidin-coated magnetic beads were treated with biotinylated small molecule ligands for 30 minutes at room temperature to generate affinity resins for kinase assays. The ligated beads were blocked with excess biotin and washed with blocking buffer to remove unbound ligand and to reduce nonspecific binding. Binding reactions were assembled by combining kinases, ligated affinity beads, and test compounds in 1× binding buffer [20% SeaBlock, 0.17 × phosphate buffered saline (PBS), 0.05% Tween 20, 6 mM DL-Dithiothreitol (DTT)]. All reactions were performed in polypropylene 384-well plates with a final volume of 20 μL. The assay plates were incubated at room temperature with shaking for 1 hour and the affinity beads were washed with wash buffer (1 × PBS, 0.05% Tween 20). The beads were then re-suspended in the elution buffer and incubated at room temperature with shaking for 30 minutes. The kinase concentration in the eluant was measured by qPCR. Two replicates were performed and averaged to obtain the Kd measurement.

### Kinome Screening Panel

For compound selectivity across a multi-kinase panel, compound binding affinity to each kinase or pseudokinase protein region was assessed using the DiscoverX scanEdge screening. Compounds were screened at a concentration of 1,000 nM in competition binding assays at Eurofins DiscoverX Corporation following the established standard protocol (DiscoverX, San Diego, CA), as described previously in the KdELECT competition binding assay. Competition with test compounds for binding to resin conjugated affinity ligand was employed to determine the “hits”, which are identified by measuring the amount of kinase captured in test versus control samples by using a quantitative PCR method that detects the associated DNA label. This measurement assesses compound selectivity against a panel of 98 kinases including kinases from the protein kinase A, G, and C family group (AGC), Ca^2+^/calmodulin-dependent protein kinase class of enzymes (CAMK), group of cyclin-dependent kinase (CDK), mitogen-activated protein kinase (MAPK), glycogen synthase kinase (GSK) and CDC-like kinase (CLK) (CMGC), Casein kinase 1 (CK1), group of Ste 7, Ste 11 and Ste 20 kinases (STE), Tyrosine Kinases (TK), Tyrosine Kinase-Like group (TKL), lipid, and atypical kinase families, and other mutant forms.

### Kit225 T Cell Assay for IFNα-Induced TYK2/phosphoSTAT1 Activity

The human Kit225 T-cell line was kindly provided by Dr. Toshiyuki Hori (Ritsumeikan University, Kyoto, Japan) and cultured in complete Roswell Park Memorial Institute (RPMI) in the presence of human interleukin 2 (IL2, R&D systems, #202-IL) following an established protocol as described in the previous literature (60, 61). Kit225 cells were seeded in 384-well plate at a density of 1×10^5^ cells/well in 4 µL Hank’s Balanced Salt Solution (HBSS), and incubated for 2 hours in a humidified, 5% CO2 cell culture incubator at 37°C. The cells were treated with serially diluted compounds for 1 hour and stimulated with 80 ng/mL human recombinant IFNα (R&D Systems) for 15 minutes. The treated cells were then lysed and cellular phosphorylated-STAT1 (Tyr701) levels were measured by AlphaLISA (PerkinElmer, Cat#ALSU-PST1) according to the manufacturer’s instructions. Inhibition data were calculated by comparison to vehicle control wells for 0% inhibition and non-stimulated control wells for 100% inhibition. Dose response curves were then generated to determine the concentration required to suppress 50% of cellular response (IC_50_) as derived by non-linear regression analysis using GraphPad Prism.

### Kit225 T Cell Assay for IL23-Induced TYK2/phosphoSTAT3 Activity

Kit225 cells were seeded in 384-well plate at a density of 1×10^5^ cells/well in 4 µL HBSS, and incubated for 2 hours in a humidified, 5% CO_2_ cell culture incubator at 37°C. The cells were treated with serially diluted compounds for 1 hour and stimulated with 100 ng/mL human recombinant IL23 (R&D Systems) for 20 minutes. The treated cells were then lysed and cellular phosphorylated-STAT3 (Tyr705) levels were measured by AlphaLISA (PerkinElmer, Cat# ALSU-PST3) according to the manufacturer’s instructions. Inhibition data were calculated by comparison to vehicle control wells for 0% inhibition and non-stimulated control wells for 100% inhibition. Dose response curves were then generated to determine the concentration required to suppress 50% of cellular response (IC_50_) as derived by non-linear regression analysis using GraphPad Prism.

### Jurkat T Cell Reporter Assay for IFNα Induced TYK2-Mediated IRF Activity

Jurkat-Dual™ T cells (*In vivo*gen, jktd-isnf) were purchased from *In vivo*gen (San Diego, CA). The cells feature the dual-reporter system, with a Lucia luciferase gene driven by an interferon-β (IFN-β) minimal promoter fused to the NFκB consensus transcriptional response element and the c-Rel binding site, as well as a secreted embryonic alkaline phosphatase (SEAP) reporter gene under the control of an IFN-stimulated gene 54 (ISG54) minimal promoter in conjunction with five IFN-stimulated response elements. With the dual-reporter design, Jurkat-Dual™ cells allow the simultaneous investigation of both the NFκB pathway, by monitoring the Lucia luciferase, and the interferon regulatory factor (IRF) pathway, by assessing the activity of SEAP. In this study, the Jurkat-Dual™ T cells were plated at a sterilized U-shaped 96-well plate in RPMI (Thermo Fisher) containing 10% heat-inactivated Fetal Bovine Serum (FBS) (Thermo Fisher) and 100 U/mL Penicillin-Streptomycin (Thermo Fisher) at a density of 2.0 × 10^6^/mL. Seeded cells were treated with serially diluted compounds and stimulated with 80 ng/mL human recombinant IFNα (Biolegend, # 592702) overnight in a humidified, 5% CO2 cell culture incubator at 37°C. Activity of SEAP was then detected from the cell cultured supernatant according to the manufacturer’s instructions. Inhibition data were calculated by comparison to vehicle control wells for 0% inhibition and non-stimulated control wells for 100% inhibition. Dose response curves were then generated to determine the concentration required to suppress 50% of cellular response (IC_50_) as derived by non-linear regression analysis using GraphPad Prism.

### IFNα-Induced CXCL10 Production in Human Whole Blood Specimens

Human whole blood [drawn with Ethylenediaminetetraacetic acid (EDTA) as anti-coagulant] was obtained from Zen-Bio Inc. (Durham, NC). Blood samples were incubated with compounds in a sterilized U-shaped 96-well plate with a volume of 200 μL/well, and stimulated with 80 ng/mL recombinant human IFNα (BioLegend, #592706) for 16 hours in a humidified, 5% CO2 cell culture incubator at 37°C. The plasma was collected for detection of C-X-C motif chemokine 10 (CXCL10, IP10) secretion. CXCL10 was measured by Human CXCL10/IP-10 AlphaLISA Kit (PerkinElmer, #AL259 C/F) according to the manufacturer’s instructions. Inhibition data were calculated by comparison to vehicle control wells for 0% inhibition and non-stimulated control wells for 100% inhibition. Dose response curves were then generated to determine the concentration required to suppress 50% of cellular response (IC_50_) as derived by non-linear regression analysis using GraphPad Prism.

### IL12-Induced IFNγ Production in Human Peripheral Blood Mononuclear Cells (PBMCs)

Human PBMC samples were obtained from iQ Biosciences, CA. 1.0 × 10^6^/mL PBMCs were seeded in complete RPMI at a sterilized U-shaped 96-well plate with a volume of 200 μL/well. PBMCs were treated by serially-diluted test compounds, and incubated with 40 ng/mL recombinant human IL12 (Biolegend, #573002) for overnight in a humidified, 5% CO_2_ cell culture incubator at 37°C. The supernatant was then collected for detection of IFNγ secretion. IFNγ level was measured by Human IFNγ AlphaLISA Kit (PerkinElmer, AL217C/F) according to the manufacturer’s instructions. Inhibition data were calculated by comparison to vehicle control wells for 0% inhibition and non-stimulated control wells for 100% inhibition. Dose response curves were then generated to determine the concentration required to suppress 50% of cellular response (IC_50_) as derived by non-linear regression analysis using GraphPad Prism.

### IFNα-Stimulated STAT5 Phosphorylation in Human Whole Blood

Human whole blood (drawn with EDTA as anti-coagulant) was obtained from Zen-Bio Inc. (Durham, NC). Whole blood samples were seeded at a sterilized U-shaped 96-well plate with a volume of 200 μL/well. After one-hour incubation with serially-diluted test compounds, whole blood samples were stimulated with 80 ng/mL recombinant human IFNα (BioLegend, #592706) for 15 minutes, in a humidified, 5% CO_2_ cell culture incubator at 37°C. Blood was lysed for the removal of red blood cells and fixed with Fix/Lyse buffer (BD 558049), washed, and permeabilized on ice using Perm III buffer (BD 558050). Cells were stained with anti-CD3 FITC antibody (BD 555916), AF647 anti-Stat5 (pY694) antibody (BD 612567) for 30 minutes on ice. Samples were then analyzed by flow cytometry using the Guava^®^ EasyCyte Instrument (EMD Millipore, Burlington, MA). Phosphorylation of cellular STAT5 was quantitated by median fluorescence intensity (MFI) after gating on the CD3-positive population.

### JAK Kinase Inhibition Assays

The biochemical JAK (JAK1, JAK2, JAK3 and TYK2) kinase activity measurement was performed using Z’LYTE™ kinase assay kit (Cat# PV4122, ThermoFisher Scientific, Madison, WI). Serially diluted test compounds were screened in 1% DMSO (final) and added into a kinase reaction buffer in a 384 well-plate (Corning Cat. # 3676) for kinase reaction. The 10 μL kinase reaction mixture contains 1× inhibitor, 1× Kinase, 1× ATP, and 2 μM Z9-LYTE™ Tyr6 Peptide. After 1-hour incubation at room temperature, 5 μL development solution was added into each reaction and incubated for 1-hour at room temperature. Next, 5 μL stop solution was then added into the final reaction. Finally, the fluorescence signals were measured on a PHERAstar microplate reader (BMG LABTECH, Germany). The curve fit was generated, and statistical analysis was performed using GraphPad Prism. IC_50_ values were derived by non-linear regression analysis and defined as the compound concentration at which the response level was reduced to half of its maximum relative to a DMSO control.

### JAK Cellular Selectivity Assays

JAK reference cell assays for JAK1 [interleukin 6 (IL6)-induced phospho-STAT3 (Tyr705)], JAK2 [Erythropoietin (EPO)-induced phospho-STAT5 (Tyr694/699)], JAK1/3 [IL2-induced phospho-STAT5 (Tyr694/699)] were performed to assess compound for JAK cellular functional selectivity. Briefly, human TF1 erythroblasts (ATCC CRL-2003) or Kit225 cells were seeded in 384-well plate at a density of 5 ×10^4^ cells/well in 4 µL HBSS. The cells were then ready for the assay after incubating for 2 hours in a humidified, 5% CO_2_ cell culture incubator at 37°C. Serially diluted compounds were added to 5 ×10^4^ cells in RPMI 1640 per well in 384-well microtiter plates, and incubated for 1 hour before the addition of activating cytokines (40 ng/mL human recombinant IL6, 100 ng/mL human recombinant EPO, or 20 ng/mL human recombinant IL2). Cytokines were purchased from the following sources: IL6 (Biolegend), EPO (R&D Systems) and IL2 (Biolegend), and were used at final concentrations that stimulated 50–90% of the maximal cytokine-induced signal in each assay (i.e., EC_50–90_ of the cytokine for each assay readout). Phosphorylation of STAT3 [(Tyr705), IL6-induced-TF1], STAT5 [(Tyr694/699), EPO-induced-TF1], or STAT5 [(Tyr694/699), IL2-induced-Kit225] was measured in cell lysates using AlphaLISA SureFire Ultra pSTAT Kits (Perkin Elmer, MA) using the manufacturer’s protocol. The signal was read on PHERAstar FSX instrument, using AlphaLISA settings (680 nm/615 nm) for STAT phosphorylation. The curve fit was generated, and statistical analysis was performed using GraphPad Prism. IC_50_ values were derived by non-linear regression analysis and defined as the compound concentration at which the response level was reduced to half of its maximum relative to a DMSO control.

### Molecular Modelling

The JAK2 JH2 modeled complexes were generated using the protein coordinates from Protein Data Bank (PDB) ID: 5UT3 (55). Acetyl groups were added to the N-termini and the C-termini were amidated after removing the compound (IKK-2 Inhibitor VI). Parameter and topology files for ligands were generated using PRODRG (62). All residues were in their default protonation states; H atoms were added and the histidines were protonated at the delta position. All crystallographic waters were included in the model. System was solvated with a box padding of 18×18×18 Å3 dimension. The modelled complexes were conjugate energy minimized using the CCP4 (version 7.1) software package (63) without applying external energy terms.

## Results

### Small-Molecule Ligands Bind With Great Affinity to the Pseudokinase JH2 Domain of TYK2

The TYK2 JH2 pseudokinase domain was reported to be catalytically inactive and serves as an allosteric autoinhibitory element that holds the JH1 kinase domain in an inactive conformation until receptor dimerization triggers the (**Figure 1**) (51–54). In this study, a series of small molecule ligands targeting the TYK2 JH2 pseudokinase domain were developed through the efforts of our drug discovery program to identify the potential therapeutic agents against TYK2-related autoimmune pathologies (**Figures 1B, C**; **Supplementary Materials and Methods**).

We performed modelling studies using the crystal structure of human JAK2 JH2 domain (PDB ID: 5UT3) complexed with potent compounds, SHR9332, 2178 and 2396 (**Figure 1**, see *Materials and Methods* for details). Modelling studies predict that compound SHR 9332 binds JH2 domain by utilizing a combination of hydrophobic and electrostatic interactions. Except for the methylated triazole ring, the remaining two aromatic rings in SHR9332 stack in a hydrophobic cleft formed by the sidechains of P694, L741, V690 and R738 (Cγ and Cδ) on one side of the cleft, and L595, V640, V603, Y689 on the other side. In addition, there are several H-bonds between the triazole ring and R738 sidechain (3.1 Å), pyridazine-3-carboxamide, and S758 and K642 sidechains. The deuteromethyl amide that was reported to provide high selectivity protrudes into a tight pocket containing A671 (**Figure 1**). The occurrence of a network of structural water molecules around the methylated triazole suggests that additional space exists for ligand extension.

Like SHR9332, compounds SHR2178 and 2396 are predicted to employ the 1H-pyrrolo [2,3-d] pyridazine core to form hydrophobic interactions in addition to a cation-pi interaction with R738. However, unlike SHR9332, the phenyl methoxy group in SHR2178 and 2396 forms an intramolecular H-bond with the amide N-H providing structural rigidity while the phenyl methoxy group in SHR9332 occupies a shallow C-terminal pocket of TYK2 JH2 in the opposite orientation. In all three compounds, the amide N-substituents (azetidine in SHR9332, and cyclopropyl in SHR2178 and 2396) extend towards a wide-open pocket.

### Small-Molecule Allosteric Ligands Achieve High Affinity-Binding for the TYK2 JH2 Pseudokinase Domain But Significantly Reduced Affinity for the Isolated TYK2 JH1 Kinase Domain

**Figure 2:**
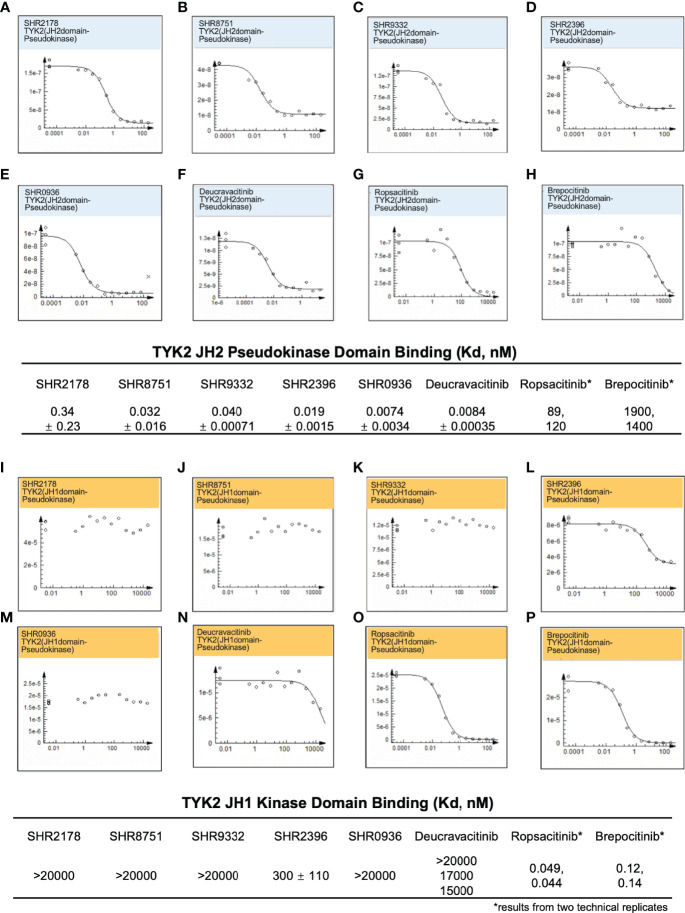
Small molecule TYK2 pseudokinase ligands showed high affinity-binding for TYK2 JH2 pseudokinase domain and low affinity for isolated TYK2 JH1 kinase domain. **(A–F)** Binding affinity of TYK2 and JAK compounds to TYK2 JH2 pseudokinase domain in KdELECT Competition Binding Assay (DiscoverX). Representative dose-response curves of: **(A)** SHR2178; **(B)** SHR8751; **(C)** SHR9332; **(D)** SHR2396; **(E)** SHR0936; **(F)** Deucravacitinib; **(G)** Ropsacitinib; **(H)** Brepocitinib. Dissociation constants (Kds) for test compound-TYK2 JH2 interactions are calculated by measuring the amount of isolated TYK2 JH2 pseudokinase captured on the solid support as function of the test compound concentrations; (I-P) Binding affinity of TYK2 and JAK compounds to TYK2 JH1 catalytic domain in DiscoverX KdELECT Competition Binding Assay. Representative dose-response curves of: **(I)** SHR2178; **(J)** SHR8751; **(K)** SHR9332; **(L)** SHR2396; **(M)** SHR0936; **(N)** Deucravacitinib; **(O)** Ropsacitinib; **(P)** Brepocitinib. Dissociation constants (Kds) for test compound-TYK2 JH1 interactions are calculated by measuring the amount of isolated TYK2 JH1 kinase captured on the solid support as function of the test compound concentrations. Confirmatory studies were performed for the compounds. The figures are representative of three independent experiments with duplicates performed for each experiment **(A–F, I–N)** unless stated otherwise. The average Kd values and standard deviations of each compound from three independent experiments are calculated and summarized in the table.

A significant obstacle in developing small-molecule TYK2 inhibitors is achieving the adequate TYK2 selectivity without affecting other JAK pathways to minimize off-target effects and the resultant undesirable toxicity. To address the selectivity issue, we employed an allosteric-based strategy and designed small molecule inhibitors (as described in **Supplementary Materials**) such as the N-cyclopropyl pyridazine carboxamide, which allosterically inhibit TYK2 JH1-kinase signaling by binding to and subsequently stabilizing the JH2 pseudokinase domain (**Figure 1**). As shown in **Figure 2**, small molecule TYK2 ligands (SHR 2178, 8751, 9332, 2396, 0936) demonstrated significant binding affinity against TYK2 JH2 pseudokinase domain with the Kds of 0.34, 0.032, 0.04, 0.029, and 0.0074 nM, respectively (**Figures 2A–E**). In contrast, all TYK2 JH2 binding ligands showed little affinity against TYK2 JH1 domain with binding Kds of >20,000, >20,000, >20,000, 300, and >20,000 nM, respectively (**Figures 2I–M**). The compounds demonstrated similar binding pattern as the clinical TYK2 allosteric inhibitor, Deucravacitinib (**Figures 2F, N**). The traditionally designed TYK2 kinase inhibitors which target the catalytic ATP binding sites (catalytic JH1 kinase domain), Ropsacitinib (PF647, TYK2-IN-8, a TYK2 kinase inhibitor) (29) and Brepocitinib (PF841, a TYK2/JAK1 inhibitor) (64, 65) only exhibited weak binding against the TYK2 JH2 domain (100 nM and 1,600 nM). They did however have a high affinity to the TYK2 JH1 domain (0.049 nM and 0.12 nM, **Figures 2G, H, Q, P**). The data demonstrate the unique binding mode of the TYK2 JH2 ligands, significantly differing from the existing ATP site targeting TYK2 kinase inhibitors.

### Small-Molecule TYK2 JH2 Pseudokinase Domain Ligands Exhibit Profound Activity in Suppressing IL23, IL12 or IFN Receptor-Mediated TYK2/STAT Phosphorylation and Cellular Functional Responses in Human Immune Cells

**Figure 3:**
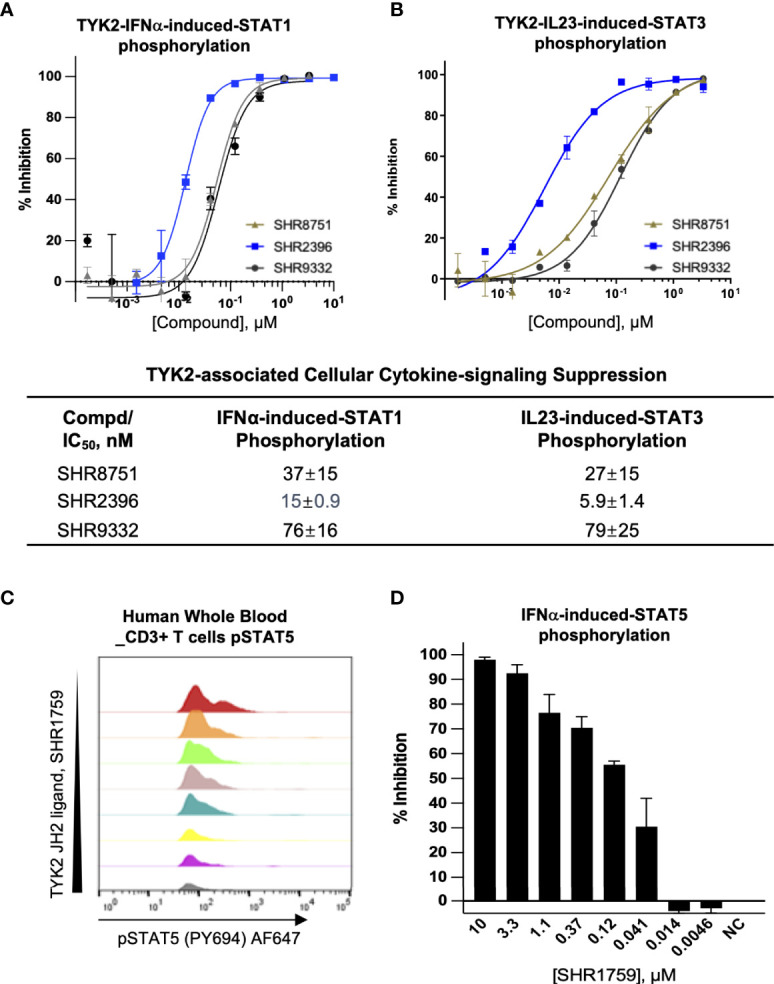
Small molecule TYK2 pseudokinase ligands block IFNα- and IL23- mediated TYK2/STAT phosphorylation in human T cells. **(A, B)** Representative dose-response curves of TYK2 JH2 ligands, SHR8751, 2396 and 9332 on IFNα-induced phosphorylation (Tyr701) of STAT1 **(A)** and IL23-induced phosphorylation (Tyr705) of STAT3 **(B)** in Kit225 cells. Cells were seeded in 384-well plate at 1×10^5^ cells/well and treated with serially diluted compounds (30 μM, 1:3) for 1 hour and stimulated with human recombinant IFNα or IL23 for 20 minutes. The treated cells were then lysed and cellular phosphorylated-STAT1 (Tyr701) or STAT3 (Tyr705) levels were measured by AlphaLISA; Inhibition data were calculated by comparison to vehicle control wells for 0% inhibition and non-stimulated control wells for 100% inhibition. Dose response curves were generated to determine the concentration required to suppress 50% of cellular response (IC_50_) as derived by non-linear regression analysis using GraphPad Prism. Confirmatory studies were performed for the compounds. The figures are representative of three independent experiments with duplicates performed for each experiment unless stated otherwise. The average IC_50_ values and standard deviations of each compound from three independent experiments are calculated and summarized in the table. **(C, D)** Suppression of STAT5 phosphorylation in human CD3+ T cells by a TYK2 JH2 ligand, SHR1759. After 1hour incubation with serially diluted compounds, human whole blood samples were stimulated with recombinant human IFNα for 15 minutes and then lysed for removal of red blood cells, fixed, and permeabilized. White blood cells were stained with anti-CD3 FITC antibody, AF647 anti-Stat5 (pY694) antibody and later analyzed by flow cytometry. **(C)** Representative histogram of AF647 showed that IFNα-induced phosphorylation of STAT5 (Tyr694) in CD3+ T cells of human whole blood was suppressed by SHR1759 in a dose-dependent manner; **(D)** After gating on the CD3-positive population, the phosphorylation of cellular STAT5 in each treatment condition was quantitated by median fluorescence intensity (MFI). Inhibition data were calculated by comparison to vehicle control wells for 0% inhibition and non-stimulated control wells for 100% inhibition. Each bar represents the % inhibition at indicated compound concentration. Confirmatory studies were performed for the compounds. The figures are representative of two independent experiments with duplicates performed for each experiment.

As an essential intracellular component of the autoimmune-related cytokine (e.g., IL23, IL12, and type I IFN) pathways, TYK2 mediates the downstream events following cytokine/receptor activation, and thus plays pivotal roles in these cytokine-associated autoimmune disorders and inflammatory pathogenesis (2, 28–32). Here, we developed the small molecule TYK2 JH2 pseudokinase ligands (SHR8751, 9332, 2396, and 1759), and demonstrated that they effectively block IFNα-induced phosphorylation of STAT1 in Kit225 T cells (**Figure 3A**) with IC_50_ values of 37 nM, 76 nM and 15 nM, respectively (SHR8751, 9332 and 2396), as well as IFNα-stimulated phosphorylation of STAT5 (Tyr694) in CD3+ T cells in human whole blood in a dose-dependent manner (SHR1759, **Figures 3C, D**). We also found that the TYK2 JH2 pseudokinase ligands (SHR8751, 9332, and 2386) suppress the IFNα down-stream IRF activity in a Jurkat Dual-reporter cell system with IC_50_ values of 48 nM, 55 nM and 9.7 nM (**Supplementary Figure 2**). In addition, the TYK2-mediated IL23-stimulated phosphorylation of STAT3 (Tyr705) in Kit225 T cells was revealed to be significantly inhibited by the TYK2 JH2 pseudokinase ligands with IC_50_ values of 27 nM, 79 nM, 5.9 nM, 24 nM and 5.0 nM (SHR8751, 9332, 2396, 3110, and Deucravacitinib, **Figure 3B** and **Supplementary Figure 6**).

**Figure 4:**
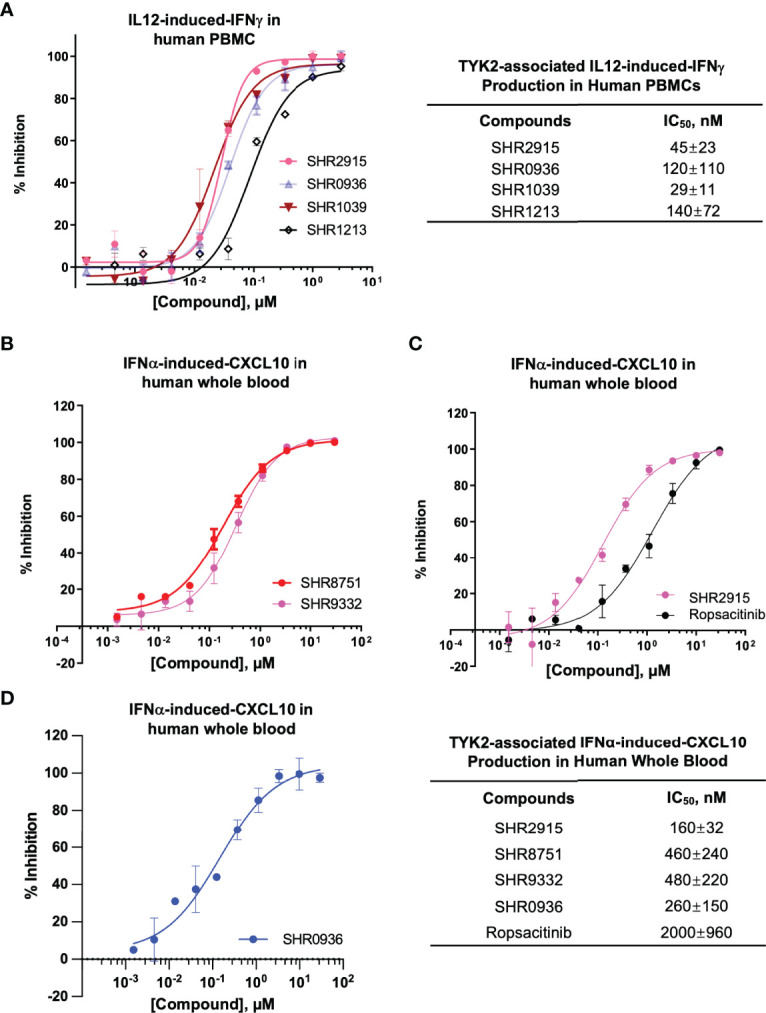
TYK2 pseudokinase ligands demonstrate significant functional activity in suppressing IL12- or IFNα- mediated TYK2/STAT pathways in human PBMCs and whole blood. **(A)** Dose dependent response of TYK2 JH2 ligands, SHR2915, 0936, 1039 and 1213 on IL12-induced IFNγ production in human PBMCs. 1.0 × 10^6^/ml PBMCs were treated by serially diluted (30 μM, 1:3) compounds and incubated with hIL12 for overnight in complete RPMI. The cultured supernatant was collected for detection of IFNγ secretion using AlphaLISA method. **(B-D)** Dose dependent effect of TYK2 JH2 ligands, SHR8751 and 9332 **(B)**, SHR2915 and Ropsacitinib **(C)** and SHR0936 **(D)** on IFNα-stimulated CXCL10 production in human whole blood. Blood samples were incubated with serially diluted compounds (30 μM, 1:3) and stimulated with recombinant human IFNα overnight. The plasma was collected for detection of CXCL10 production by AlphaLISA method. Inhibition data were calculated by comparison to vehicle control wells for 0% inhibition and non-stimulated control wells for 100% inhibition. IC_50_ values were derived by non-linear regression analysis using GraphPad Prism. The figures are representative of the experiments on specimens from three different donors with duplicates performed for each donor. The average IC_50_ values and standard deviations of each compound from experiments on three donors are calculated and summarized in the table.

Consistent with the capacity to block the cytokine-induced activation of TYK2, the new series of small molecule TYK2 pseudokinase ligands also showed equivalent potency against TYK2-mediated functional production of cell cytokines, as a critical downstream event of the immune signaling cascade. In primary human PBMCs, TYK2 pseudo-kinase ligands (SHR2915, 0936, 1039 and 1213) suppressed IL12-stimulated IFNγ production in a concentration-dependent manner with IC_50_ values of 45 nM, 120 nM, 29 nM and 140 nM, respectively (**Figure 4A**). As detailed in **Figures 4B–D**, TYK2 pseudokinase ligands (SHR2915, 8751, 9332 and 0936) also demonstrated significant potency in human whole blood against IFNα-induced CXCL10 production (**Figures 4B–D**) with IC_50_ values of 160 nM, 460 nM, 480 nM, and 260 nM. The effect is similar as the most advanced clinical TYK2 pseudokinase ligand Deucravacitinib and more potent than the TYK2 kinase inhibitor that entered Phase II clinical development against moderate-to-severe psoriasis, Ropsacitinib (**Supplementary Figure 7**; **Figures 4C**). In addition, the TYK2 JH2 ligands demonstrated broad cross-species activity revealed by the significant suppression of TYK2 cellular functional responses in the immune cells obtained from monkey, canine, rat, and mouse (**Supplementary Figure 3**). The data further supported the ability of TYK2 pseudokinase small molecule ligands to block TYK2-dependent functional cellular responses driven by IL12, IL23, or type I IFN stimulation.

### Small-Molecule TYK2 Pseudokinase Ligands Achieve Great Selectivity Among JAK Family Kinases and Reduced Off-Target JAK Functional Activities

Evidence from preclinical studies and clinical trials with JAK isoform deficiency showed that complete blockade of JAK isoforms is undesirable as it will consequently lead to severe immunodeficiency and abnormal homeostasis (15, 22, 23). Given the wide association between JAK and multiple cytokine pathways (**Supplementary Figure 1**) (23, 66–68), an important principle of JAK inhibition in that objective is not to block the pan JAK pathway completely but to selectively reduce the activity of one specific JAK isoform. One potential clinical strategy of such a mode of action is to develop a highly selective inhibitor against one JAK isoform based on its clinical relevance in certain diseases while avoiding affecting other JAK pathways non-selectively (**Supplementary Figure 1**). A notable advantage of inhibiting TYK2 by binding to its JH2 domain is the potential to achieve maximal efficacy while maintaining a high level of selectivity with respect to the other JAKs. Based upon such rationale, we designed a comprehensive biochemical and cellular analysis panel to investigate the target and cellular functional selectivity of our TYK2 pseudokinase ligands and compared them with the commercial or clinical staged TYK2 or JAK kinase inhibitors in parallel.

**Figure 5:**
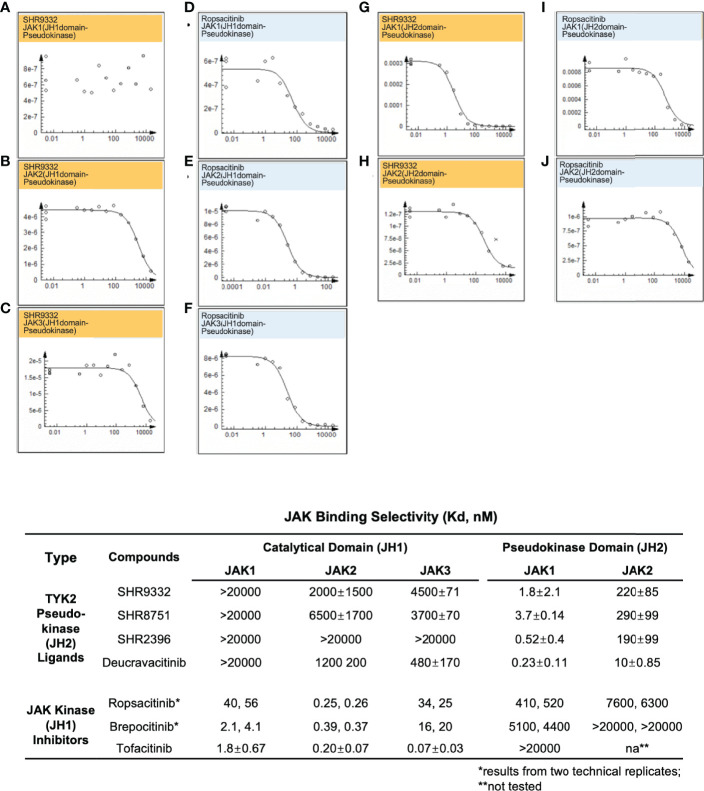
Small molecule TYK2 JH2 pseudokinase ligands demonstrate differentiated binding pattern against JAK family members compared to JH1 kinase inhibitors. **(A–C)** Representative dose-response curves for binding affinity of TYK2 JH2 ligand, SHR9332 to JH1 kinase domain of JAK1 **(A)**, JAK2 **(B)** and JAK3 **(C)** assessed by KdELECT Competition Binding Assay (DiscoverX); **(D–F)** Representative dose-response curves for binding affinity of a TYK2 kinase inhibitor, Ropsacitinib to JH1 kinase domain of JAK1 **(D)**, JAK2 **(E)**, and JAK3 **(F)** assessed by KdELECT Competition Binding Assay (DiscoverX); **(G–H)** Representative dose-response curves for binding affinity of SHR9332 to JH2 pseudokinase domain of JAK1 **(G)**, and JAK2 **(H)** assessed by KdELECT Competition Binding Assay (DiscoverX); **(I–J)** Representative dose-response curves for binding affinity of Ropsacitinib to JH2 pseudokinase domain of JAK1 **(I)** and JAK2 **(J)** assessed by KdELECT Competition Binding Assay (DiscoverX). Dissociation constants (Kds) for test compound-JH1 or -JH2 domain interactions are calculated by measuring the amount of isolated JH1 or JH2 protein segments captured on the solid support as function of the test compound concentrations. Confirmatory studies were performed for the compounds. The figures are representative of three independent experiments with duplicates performed for each experiment unless stated otherwise. The average Kd values and standard deviations of each compound from three independent experiments are calculated and summarized in the table.

In the current study, TYK2 pseudokinase ligands demonstrated significant affinity against the TYK2 JH2 domain (**Figure 2**) and high selectivity against other JAK family members (**Figure 5**). Meanwhile, these ligands had little affinity against JH1 domain across other JAK family members (JAK1, JAK2 and JAK3). As shown in **Figure 5**, the TYK2 JH2 ligand SHR9332 exhibits Kd values of greater than 20,000 nM, 2,000 nM and 4,500 nM, against the JH1 kinase domain of JAK1, JAK2 and JAK3 respectively (**Figures 5A–C**), as well as 1.8 nM and 220 nM against the JH2 domain of JAK1 and JAK2 (**Figures 5J, H**). The bindings are significantly weaker as compared to its affinity to the TYK2 JH2 (Kd = 0.04 nM, **Figure 2**). Other small molecule TYK2 pseudokinase binders demonstrated similar patterns (SHR8751, 2396, and Deucravacitinib; **Figure 5**). In contrast, the tested JAK/TYK2 kinase inhibitors (TYK2 kinase inhibitor, Ropsacitinib; TYK2/JAK1 dual inhibitor, Brepocitinib and JAK1/JAK3 inhibitor, Tofacitinib) had poor selectivity among the JAK family members (e.g., Tofacitinib Kd values among JAK1/2/3 JH1: 1.8 nM, 0.2 nM and 0.07 nM; **Figure 5**). Overall, the new series of TYK2 pseudokinase ligands showed great functional selectivity among the JAK family, which significantly differs from the ATP-site targeting JAK/TYK2 kinase inhibitors.

**Figure 6:**
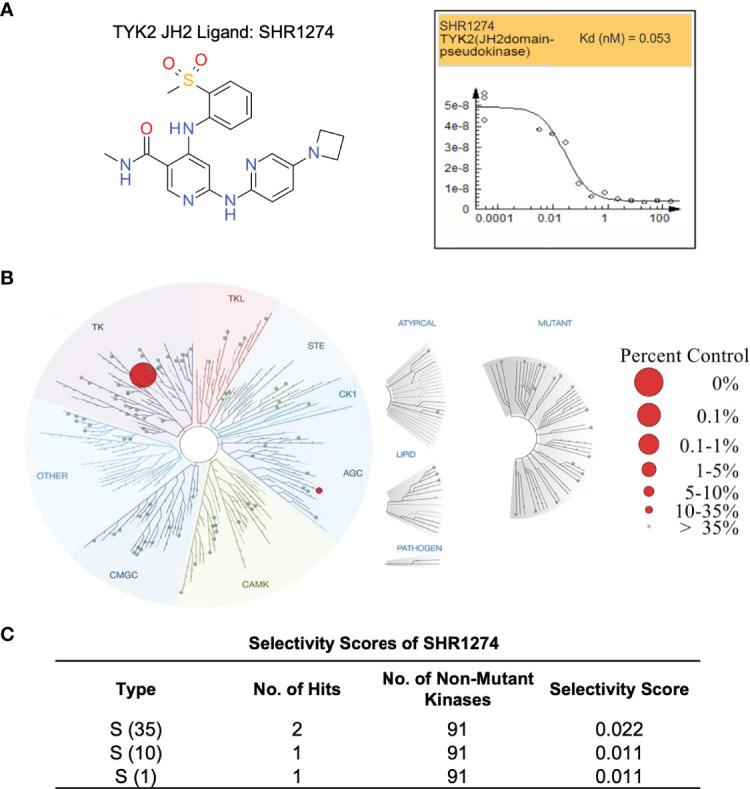
TYK2 pseudokinase small molecule ligand is highly selective across a multi-kinaseScan panel. **(A)** Chemical structure of TYK2 JH2 ligand, SHR1274 and representative dose-response curves of SHR1274 binding to TYK2 JH2 pseudokinase and JH1 kinase domains assessed by DiscoveX KdELECT Competition Binding Assay. The figures are representative of the technical duplicates in the experiment. Dissociation constants (Kds) for test compound-JH1 or -JH2 domain interactions are calculated by measuring the amount of isolated JH1 or JH2 protein segments captured on the solid support as function of the test compound concentrations. **(B)** Kinase selectivity profile of SHR1274 at 1 µM across 98 kinases in the kinome screening panel, with kinase binding inhibited by > 99% (large red circles) or 65-90% (small red circles). Screening “hits” are identified by quantifying the amount of kinase captured in test compounds versus control by using quantitative PCR that detects the associated DNA label; **(C)** Selectivity scores at S (1): > 99% competition, S (10): 90-99% competition or S (35): 65-90% competition.

The TYK2 pseudokinase ligand was also shown to be highly selective when profiled against a panel of 98 protein and lipid kinases and pseudokinases. Only TYK2 pseudokinase domain was measured to bind the small molecule ligand with Kd value of 0.053 nM (**Figure 6A**), which represents a great than 20,000-fold of selectivity over the other tested kinases in this panel (**Figures 6B, C** and **Supplementary Table 1**).

**Figure 7:**
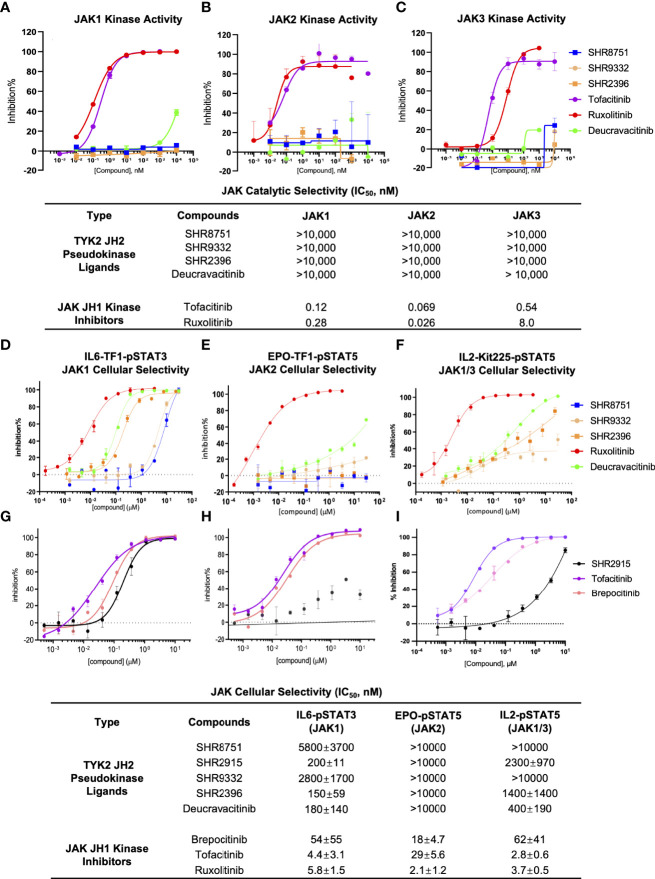
TYK2 pseudokinase ligands showed limited potency in affecting JAK1-3 functional activities. **(A–C)** Effect of TYK2 JH2 ligands SHR8751, 9332, 2396, Deucravacitinib and JAK inhibitors Tofacitinib and Ruxolitinib in suppressing JAK1 **(A)**, JAK2 **(B)** and JAK3 **(C)** kinase activities assessed by Z’LYTE kinase bio-chemical assay. Serially diluted test compounds were added into a kinase reaction buffer that contains JAK kinases (JAK1, JAK2 or JAK3, respectively) and Z9-LYTE™ Tyr6 Peptide for measurement of JAK kinase-induced peptide phosphorylation. The curve fit was generated, and statistical analysis was performed using GraphPad Prism. IC_50_ values were derived by non-linear regression analysis and defined as the compound concentration at which the response level was reduced to half of its maximum relative to a DMSO control; **(D, G)** Effect of TYK2 JH2 ligands and TYK2/JAK kinase inhibitors SHR8751, 9332, 2396, Deucravacitinib and Ruxolitinib **(D)** and SHR2915, PF841 and Tofacitinib **(G)** on JAK1-dependent IL6-induced phosphorylation of STAT3 (Tyr705) in TF1 cells. TF1 cells were treated by serially diluted compounds (30 μM, 1:3) for 1 hour and stimulated by recombinant human IL6 for 20 mins. Phosphorylation of STAT3 (Tyr705) was measured in cell lysates using AlphaLISA assay; **(E, H)** Effect of TYK2 JH2 ligands and TYK2/JAK kinase inhibitors SHR8751, 9332, 2396, Deucravacitinib and Ruxolitinib **(E)** and SHR2915, PF841 and Tofacitinib **(H)** on JAK2-dependent EPO-induced phosphorylation of STAT5 (Tyr694/699) in TF1 cells. TF1 cells were treated by serially diluted compounds (30 μM, 1:3) for 1 hour and stimulated by recombinant human EPO for 20 mins. Phosphorylation of STAT5 (Tyr694/699) was measured in cell lysates using AlphaLISA assay; **(F, I)** Effect of TYK2 JH2 ligands and TYK2/JAK kinase inhibitors SHR8751, 9332, 2396, Deucravacitinib and Ruxolitinib **(F)** and SHR2915, PF841 and Tofacitinib **(I)**, on JAK1/3-dependent IL2-induced phosphorylation of STAT5 (Tyr694/699) in Kit225 cells. Kit225 cells were treated by serially diluted compounds (30 μM, 1:3) for 1 hour and stimulated by recombinant human IL2 for 20 mins. Phosphorylation of STAT5 (Tyr694/699) was measured in cell lysates using AlphaLISA assay. For each assay, inhibition data were calculated by comparison to vehicle control wells for 0% inhibition and non-stimulated control wells for 100% inhibition. Dose response curves were generated to determine the IC_50_ values as derived by non-linear regression analysis using GraphPad Prism. Confirmatory studies were performed for the compounds. The figures are representative of three independent experiments with duplicates performed for each experiment unless stated otherwise. The average IC_50_ values and standard deviations of each compound from three independent experiments are calculated and summarized in the table.

The selectivity of TYK2 pseudokinse ligands was also measured by JAK kinase functional phosphorylation assays. All our TYK2 JH2 compounds were demonstrated to be very selective and showed little activity with the IC_50_ of greater than 10,000 nM in all JAK kinase assays (JAK1, JAK2, and JAK3) (**Figures 7A–C**). However, the JAK1/JAK3 kinase inhibitor, Tofacitinib, and a pan JAK inhibitor, Ruxolitinib, were demonstrated to be very potent in blocking all JAK kinase activities with poor selectivity. Tofacinitib showed IC_50_ values of 0.12 nM, 0.069 nM, and 0.54 nM against catalytic activities of JAK1, 2 and 3, and Ruxolitinib had IC_50_ values of 0.28 nM, 0.026 nM, and 8.0 nM, respectively (**Figures 7A–C**).

Consistent with the significant biochemical selectivity among JAK family members, TYK2 pseudokinase ligands also considerably lack potency in a panel of JAK1/2/3-dependent cellular functional assays (**Figures 7D–I**). All tested TYK2 JH2 ligands (SHR8751, 2915, 9332, 2396, and Deucravacitinib) showed exceptional selectivity with IC_50_ of >10,000 nM against the JAK2 dependent EPO-stimulated phosphorylation of STAT5 (Tyr694/699) in Kit225 T cells, whereas the traditional JAK and TYK2 kinase inhibitors (Brepocitinib, Tofacitinib and Ruxolitinib) were quite potent in JAK2 dependent cellular functional assay with IC_50_ values of 54 nM, 4.4 nM and 5.8 nM (**Figures 7E, H**). In the JAK1/3 dependent IL2-stimulated phosphorylation of STAT5 measurement, our TYK2 JH2 ligands also demonstrated great selectivity with IC_50_ values of >10,000 nM, and >1,000 nM, representing over >200 or >20 less potent inhibition than was measured against TYK2-dependent function in the same cellular settings (**Figures 3A, B**; **7F, I**). In addition, the functional suppression of JAK1 dependent IL6-stimulated STAT3 (Tyr705) phosphorylation by TYK2 JH2 ligands was also considerably weaker than that was tested against TYK2 dependent STAT3 (Tyr705) phosphorylation (**Figures 3B**; **7D, G**). The selectivity profile of TYK2 JH2 pseudokinase ligands contrasts greatly with all other JAK kinase inhibitors (**Figures 1**, **5**, **7**; **Supplementary Figures 4, 5**). The DMPK profile was also documented for the further development of the TYK2 JH2 pseudokinase ligands (**Supplementary Table 2**). The profound potency and high selectivity of TYK2 JH2 small molecule ligands support endeavors to develop clinically effective TYK2 inhibitors with a potentially well-tolerated safety profile for autoimmune disease therapeutics.

## Discussion

Several JAK inhibitors such as Ruxolitinib, Tofacitinib, Baricitinib, and Upadacitinib have been approved for the treatment of chronic inflammatory and myeloproliferative diseases (67–72). However, the clinical benefit of these JAK inhibitors had been largely restricted due to the potential safety risks. The typical safety issues associated with JAK kinase inhibitors include infections and adversely affected laboratory values, such as neutropenia, changes in hemoglobin, HDL and serum creatinine levels, as well as thromboembolic events (15, 21, 22). In fact, in September 2021, the FDA announced that Xeljanz (Tofacitinib), Xeljanz XR, Oluminant (Baricitinib), and Rinvoq (Upadacitinib) will be required to have updated warnings about an increased risk of heart-related events such as heart attack or stroke, cancer, blood clots, and death (17–20).

In the past decade, TYK2 became an emerging drug target to treat various human autoimmune diseases. A selective TYK2 inhibitor offers a great opportunity to treat autoimmune diseases delivering a potentially differentiated clinical profile compared to currently approved JAK inhibitors (25, 27, 28, 35). However, due to the high homology in the catalytic domain among JAK family members, discovery of selective TYK2 inhibitor over other JAKs by the traditional method of targeting the catalytically active site (JH1 domain) remains challenging (27, 52). In this study, we employed a strategy to develop a series of small molecule TYK2 pseudokinase ligands, which specifically bind to the TYK2 JH2 regulatory domain of the TYK2 protein and dispense the TYK2 JH1 catalytic domain. The TYK2 pseudokinase ligands inhibit the TYK2 catalytic activity in an allosteric manner and restrict the conformational mobility of the JH1 catalytic sites which is required for phosphorylation. Our characterizations demonstrated the compounds in this study are highly potent TYK2 pseudokinase binders and greatly selective against other JAK family members (JAK1, JAK2, and JAK3). Their selectivity profile is positively differentiated as compared to the traditionally-designed JAK and TYK2 kinase inhibitors which target the ATP binding site (**Figure 5**; **Supplementary Figures 1, 5**).

We investigated the cellular functional activities of the selective TYK2 JH2 ligands in the TYK2-mediated cytokine-driven cellular pathways (IFNα, IL12 and IL23). A series of TYK2 JH2 ligands (SHR2178, 9332, 8751, 2396, 0936, 3110, 3118, etc.) showed a significant ability to block several TYK2-mediated cytokine-stimulated pathway activities in immune cells, including IFNα-induced STAT1 (Tyr701) phosphorylation, IFNα-IRF activities, IL23-mediated-STAT phosphorylation, IL12-driven-IFNγ production, and IFNα-stimulated-CXCL10 production in human immune cells (**Figures 2**–**4**; **Supplementary Figures 2, 6, 7**).

Targeting the JH2 pseudokinase domain of TYK2 rather than the active site of the JH1 catalytic kinase domain represents a unique approach with greatly improved selectivity as compared to the traditionally designed kinase inhibitors. As stated above, the TYK2 pseudokinase ligands act allosterically to suppress the cytokine-driven activation of the TYK2 kinase, which demonstrated great potency in blocking TYK2 dependent cellular signal transduction without significantly affecting the JAK-related cellular cytokine-functional pathways. As a result, targeting the pseudokinase of TYK2 offers a promising path for the development of highly selective TYK2 inhibitors. These developments support the investigation of a wide dose range in clinical trials without elevating the risk of off-target toxicity owing to other related kinase activities, particularly the JAK family kinases. Traditionally designed JAK kinase inhibitors that target JAK catalytic site, such as Ruxolitinib (pan JAK kinase inhibitor) (16, 68) and Tofacitinib (JAK1/JAK3 dual inhibitor with little selectivity over JAK2) (69, 70) were designed to target the ATP binding site in catalytic domain and show poor selectivity within the JAK family (16, 21, 27) (**Supplementary Figure 1**). This is largely due to the nature of the high homology within the active site of JAK kinase family. It has been known that the JAK kinases, JAK1, JAK2, and JAK3 regulate signal transduction for more than 20 pathways driven by cytokines and growth factors (**Supplementary Figure 1**) (14, 23, 24, 66). Pan-JAK inhibitors were revealed to have limited therapeutic margins owing to the increased risks of anemia, leukopenia and infection during treatment (17–21). So far, no clinical agents for the JH1 catalytic site of TYK2 have been reported to be selective despite considerable medicinal chemistry efforts, including Ropsacitinib (TYK2 kinase inhibitor) and Brepocitinib (TYK2/JAK1 dual inhibitor) that both entered Phase II clinical trial development against moderate to severe plaque psoriasis and other auto-immune disorders as orally administered drugs (NCT03895372, NCT02969018, NCT05076006, etc.) (29, 64, 65). In the current study, the side-by-side comparison between the small molecule TYK2 JH2 pseudokinse ligands (SHR2396, 8751, 9332 and 2915) and the above stated TYK2 active-site-directed inhibitors (Ropsacitinib and Brepocitinib) demonstrated the advantages of TYK2 pseudokinase ligands in both potency and selectivity (**Figures 5**, **7**). Ropsacitinib and Brepocitinib were found to exhibit varying degrees of JAK 1/2/3 functional inhibition. The results are consistent with the previous studies, which suggest a lack of meaningful differences inhibition profiles among the JAK family members (27, 56, 57). Nevertheless, the absolute IC_50_ values from the current study may not be directly comparable with some of other studies, likely due to the utilization of different assay systems and readouts, as well as other differences in lab conditions, personnel, or laboratory facilitates (29, 71, 72).

A breakthrough TYK2 allosteric inhibitor, Deucravacitinib (BMS986165), is in late-stage clinical development for multiple autoimmune diseases. Deucravacitinib achieves a high degree of selectivity by binding to the JH2 regulatory domain of TYK2 (27, 52, 56–59), which allows the compound to effectively inhibit TYK2 and does not affect JAK1, JAK2 or JAK3 pathways at therapeutic doses. In a phase II clinical trial against moderate to severe psoriasis involving 267 patients, Deucravacitinib was reported to be clinically effective at a daily dose of 3 mg and above (NCT02931838) (58). The most common adverse events (nasopharyngitis, headache, diarrhea, nausea and upper respiratory tract infection) occurred slightly more often in Deucravacitinib treated patients compared to the placebo group (55-80% vs 51%). None of the serious adverse events reported in the treatment group was assessed as being drug-related (27, 52, 56–59). The impressive clinical efficacy and safety profile of the TYK2 JH2 compound, Deucravacitinib, further support the notion of designing the allosteric TYK2 pseudokinase small-molecule ligands to achieve great selectivity for an optimal therapeutic window. In this study, the newly designed TYK2 JH2 ligands (SHR2915, 0936, 9332, 1039, 1213 and 3110) demonstrated similar potency and selectivity as compared to Deucravacitinib (BMS986165) (**Supplementary Figures 5–7**). The current data are similar to the *in vitro* results of Deucravacitinib which were previously reported (27, 56).

In conclusion, this study supported the promising therapeutic potential of targeting TYK2 JH2 pseudokinase with small-molecule ligands that allosterically mediate the JH2-JH1 regulation to block the cytokine-induced activation of TYK2, in the development of therapeutics for multiple cytokine-driven autoimmune diseases. Unlike the traditionally designed kinase inhibitors targeting ATP binding sites of JAK and TYK2, which are undesirably less selective among members of the JAK family (21, 22, 27), the TYK2 JH2 ligands employ a differentiated mechanism of action *via* binding to the pseudokinase domain for selectively suppressing TYK2 without affecting other JAK functional pathways.

## Data Availability Statement

The original contributions presented in the study are included in the article/**Supplementary Material**. Further inquiries can be directed to the corresponding author.

## Author Contributions

YZ, XL, and XW designed the experiments; RS, YZ, FZ, and DL performed biochemical or cellular assays; YZ, RS, FZ, and DL conducted data analyses; XL, XW, JLiu, PL, YY, PD, ZZ, and HW synthesized compounds; RC, MM, and MI conducted molecular modeling; YZ and XL wrote the manuscript with contributions from WT, FH, CB, JLi, JF, HC, YM, SL, and LZ; All authors contributed to data generation and reviewed the manuscript. All authors contributed to the article and approved the submitted version.

## Conflict of Interest

YZ, RS, XW, FZ, DL, JLiu, SL, YY, HW, JLi, PL, LZ, RC, MM, and MI were employees of Eternity Bioscience Inc. XL, PD, ZZ, YM, JF, HC, CB, FH, and WT are employees and shareholders of Hengrui Pharmaceutical Co. Ltd.

## Publisher’s Note

All claims expressed in this article are solely those of the authors and do not necessarily represent those of their affiliated organizations, or those of the publisher, the editors and the reviewers. Any product that may be evaluated in this article, or claim that may be made by its manufacturer, is not guaranteed or endorsed by the publisher.
