# Supplementary material for: Novel Small Molecule Tyrosine Kinase 2 Pseudokinase Ligands Block Cytokine-Induced TYK2-Mediated Signaling Pathways
Source: Front Immunol. 2022 May 20;13:884399. doi: 10.3389/fimmu.2022.884399 (PMC9186491; doi:10.3389/fimmu.2022.884399)

Supplementary Materials for

Small Molecule Tyrosine Kinase 2 (TYK2) Pseudokinase Ligands Block Cytokine-induced TYK2-mediated Signaling Pathways

Yu Zhou[1*+], Xin Li[2*+], Ru Shen[1], Xiangzhu Wang[1], Fan Zhang[1], Suxing Liu[1], Di Li[1], Jian Liu[1], Puhui Li[1], Yinfa Yan[1], Ping Dong[2], Zhigao Zhang[2], Heping Wu[1], Linghang Zhuang[1], Rasheduzzaman Chowdhury[1], Matthew Miller[1], Mena Issa[1], Yuchang Mao[2], Hongli Chen[2], Jun Feng[2], Jing Li[1], Chang Bai[2], Feng He[2], and Weikang Tao[2]

*Corresponding author: Yu Zhou, E-mail: yuzhou8200@gmail.com;
Xin Li, E-mail: xin.li.xl6@hengrui.com
[+]these authors contributed equally

The file includes:

Compound Synthetic Procedures

Supplementary Figure 1. TYK2/JAK-mediated cytokine signaling pathways and therapeutic TYK2/JAK inhibitors

Supplementary Figure 2. Activities of TYK2 JH2 ligands to suppress IFNα-induced-TYK2-associated signaling pathway

Supplementary Figure 3. TYK2 JH2 compounds showed broad cross-species activities (Monkey, Canine, Rat and Mouse)

Supplementary Figure 4. TYK2 JH1/JH2 binding for Tofacitinib

Supplementary Figure 5. JAK1/2/3, TYK2 JH1/JH2 binding for BMS165 (Deucravacitinib), PF841 (Brepocitinib), PF647 (Ropsacitinib), SHR0936, 2915, 1039, and 1213

Supplementary Figure 6. Inhibition of IFNα- or IL23- induced TYK2-associated TYK2-mediated cellular activities by JAK/TYK2 inhibitors, SHR3110, Filgotinib, Baricitinib, Upadacitinib, Ruxolitinib, and Deucravacitinib

Supplementary Figure 7. Suppression of IFNα-induced TYK2-associated functional activities in human whole blood by TYK2/JAK inhibitors, SHR3110, Filgotinib, Baricitinib, Upadacitinib, Ruxolitinib, and Deucravacitinib

Supplementary Table 1. KINOMEscanEdge profiling for SHR1274

Supplementary Table 2. DMPK profile of SHR2396, 8751, 9332, 2178, 4995, and 4997

Compound Synthetic Procedures

4-(cyclopropanecarboxamido)-2-(2-methoxyphenyl)-N-methyl-1H-pyrrolo[2,3-d]pyridazine-7-carboxamide (SHR2178)

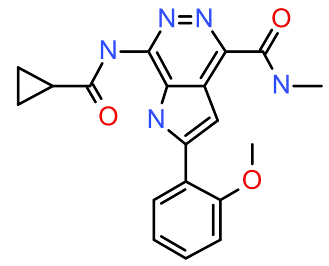

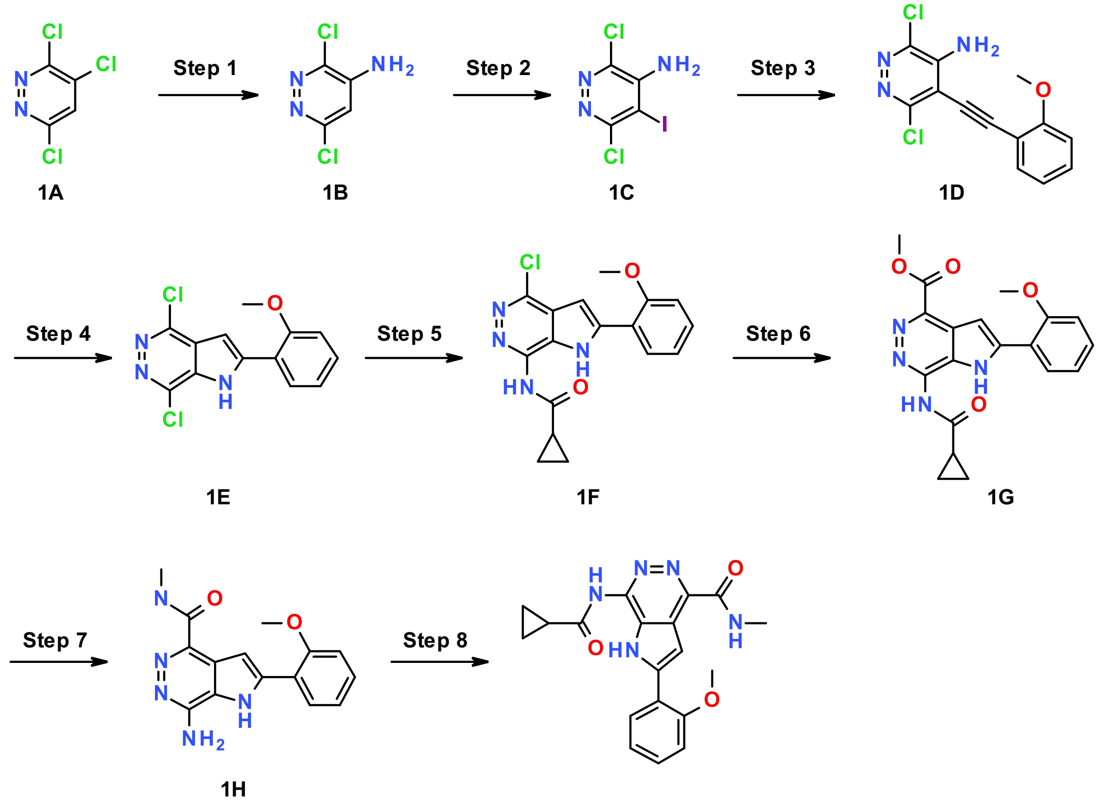

Step 1

3,6-dichloropyridazin-4-amine

3,4,6-trichloropyridazine (100 g, 545.19 mmol, Bidepharm) was mixed with NH_3/H_2O (2500 mL). The mixture was sealed and stirred at 85°C for 16 hours. The mixture was cooled to room temperature and filtered to give 3,6-dichloropyridazin-4-amine (59.5 g, 362.8 mmol, yield: 66.5%) as a white solid.

LCMS: MS m/z (ESI): 164.0 $[M+H]^+$

Step 2

3,6-dichloro-5-iodopyridazin-4-amine

To a solution of 2,2,6,6-tetramethylpiperdine (64.60 g, 457.3 mmol, Bidepharm) in THF (1000 mL) n-butyllithium (243.9 mL, 2.5 M in hexane, Bidepharm) was added at -78°C. The mixture was stirred for 10 min, then 3,6-dichloropyridazin-4-amine (25 g, 152.4 mmol) was added. After stirring for additional 30 min, I_2 (42.56 g, 167.7 mmol, Sinopharm) was added. The mixture was maintained at -78°C for additional 2h and then quenched with water. The reaction mixture was acidified by aqueous HCl (6N) and subsequently extracted with Ethyl Acetate. The combined ethyl acetate was dried over Na_2SO_4 and filtered. The filtrate was concentrated, and the resulting crude was purified by silica gel chromatography (PE: EA=4:1) to afford the title compound (11 g, 37.9 mmol, yield: 24.9%) as a yellow solid.

LCMS: MS m/z (ESI): 289.8 $[M+H]^+$

Step 3

3,6-dichloro-5-((2-methoxyphenyl)ethynyl)pyridazin-4-amine

The mixture of 3,6-dichloro-5-iodopyridazin-4-amine (11 g, 37.95 mmol), 1-ethynyl-2-methoxy-benzene (5.52 g, 41.74 mmol, Bidepharm), CuI (722.67 mg, 3.79 mmol) and $PdCl_2(PPh_3)_2$ (2.66 g, 3.79 mmol, greenchem) in Et_3N (10.55 mL, 75.89 mmol, energy-chemical) was stirred at r.t. under Ar for 16 h. The mixture was poured into water, and the resulting solid was collected filtered and washed with water. The crude solid was recrystallized with EA and PE to afford desire product (6 g, 20.40 mmol, yield: 53.76%) as a yellow solid.

LCMS: MS m/z (ESI): 294.0 $[M+H]^+$

Step 4

4,7-dichloro-2-(2-methoxyphenyl)-1H-pyrrolo[2,3-d]pyridazine

The mixture of 3,6-dichloro-5-((2-methoxyphenyl)ethynyl)pyridazin-4-amine (6 g, 20.40 mmol) and t-BuOK (9.16 g, 81.59 mmol, Sinopharm) in THF was stirred at 90°C for 16 h. The mixture was quenched with water, extracted with Ethyl Acetate. The organic solution was washed with brine, dried over Na_2SO_4 and then filtered. The filtrate was concentrated under vacuum, the resulting residue was purified by silica gel chromatography (PE:EA=2:1) to afford title compound (1.2 g, 4.08 mmol, yield: 20.0%) as a yellow solid.

LCMS: MS m/z (ESI): 293.9 $[M+H]^+$

Step 5

N-(7-chloro-2-(2-methoxyphenyl)-1H-pyrrolo[2,3-d]pyridazin-4-yl)cyclopropanecarboxamide

The mixture of 4,7-dichloro-2-(2-methoxyphenyl)-1H-pyrrolo[2,3-d]pyridazine (1.2 g, 4.08 mmol), cyclopropanecarboxamide (347.20 mg, 4.08 mmol, Bidepharm) and Cs_2CO_3 (1.99 g, 6.12 mmol, Sinopharm) in Dioxane (20 mL) was bubbled with nitrogen for 5 minutes, Xantphos

(944.25 mg, 1.63 mmol, Bidepharm) and $Pd_2(dba)_3$ (747.18 mg, 815.95 umol, zhejiangyejin) were added under N_2 atomosphere. The reaction tube was sealed and heated to 130°C for 16h. The mixture was cooled to room temperature, and diluted with DCM. The organic solution was washed with brine, dried over Na_2SO_4 and then filtered. The filtrate was concentrated under vacuum, the resulting residue was purified by silica gel chromatography (DCM:EA=1:4) to afford desire product (320 mg, 933.55 umol, yield: 22.9%) as a yellow solid.

LCMS: MS m/z (ESI): 343.0 $[M+H]^+$

Step 6

Methyl 4-(cyclopropanecarboxamido)-2-(2-methoxyphenyl)-1H-pyrrolo[2,3-d]pyridazine-7-carboxylate

The mixture of N-(7-chloro-2-(2-methoxyphenyl)-1H-pyrrolo[2,3-d]pyridazin-4-yl)cyclopropanecarboxamide (170 mg, 0.5mmol), $Pd(dppf)Cl_2$ (18mg, 0.025 mmol, zhejiangyejin) and Et_3N (0.21mL, 1.49 mmol, energy-chemical) in MeOH (50 mL) were heated to 100°C under CO atmosphere (5 atm pressure) for 16 hours. The reaction mixture was cooled to rt and concentrated, the crude residue was purified by silica gel chromatography (DCM:EA=1:2) to afford title compound (120 mg, 0.33mmol, yield:66.0%) as a yellow solid.

LCMS: MS m/z (ESI): 367.0 $[M+H]^+$

Step 7

4-amino-2-(2-methoxyphenyl)-N-methyl-1H-pyrrolo[2,3-d]pyridazine-7-carboxamide

Methyl 4-(cyclopropanecarboxamido)-2-(2-methoxyphenyl)-1H-pyrrolo[2,3-d]pyridazine-7-carboxylate (120 mg, 0.33mmol) was dissolved in methylamine/MeOH (2M, 20 mL,

Sinopharm). The mixture was sealed in reaction tube and heated at 90°C for 16h. The mixture was cooled to rt and then concentrated to afford desire product (95 mg, 0.32mmol, yield: 97.5%) as a yellow solid without further purification.

LCMS: MS m/z (ESI): 298.0 $[M+H]^+$

Step 8

4-(cyclopropanecarboxamido)-2-(2-methoxyphenyl)-N-methyl-1H-pyrrolo[2,3-d]pyridazine-7-carboxamide

The mixture of cyclopropanecarboxylic acid (38.21 uL, 0.48 mmol, Bidepharm) DIEA (154.86 uL, 0.96mmol, danyu), Bop (130.45 mg, 0.64mmol, Bidepharm) and 4-amino-2-(2-methoxyphenyl)-N-methyl-1H-pyrrolo[2,3-d]pyridazine-7-carboxamide (95 mg, 0.32mmol) was stirred at r.t. for 16 h. The mixture was diluted with DCM and washed with brine, dried over Na_2SO_4 and then filtered. The filtrate was concentrated under vacuum, the resulting residue was purified by silica gel chromatography (PE:EA=1:1) to afford 50 mg of title compound (yield: 42.83%) as a white solid.

^{1}H NMR (400 MHz, DMSO-d_6): δ 12.62 (s, 1H), 11.72 (s, 1H), 8.89 (d, J = 4.8 Hz, 1H), 8.01 (dd, J = 8.0 Hz, 1.6 Hz, 1H), 7.62 (d, J = 2.0 Hz, 1H), 7.49-7.44 (m, 1H), 7.26 (d, J = 8.0 Hz, 1H), 7.16-7.11 (m, 1H), 4.01 (s, 3H), 2.89 (d, J = 4.8 Hz, 3H), 2.34-2.30 (m, 1H), 1.05-0.97 (m, 4H). LCMS: MS m/z (ESI):366.0 $[M+H]^+$

6-((5-(azetidin-1-yl)pyridin-2-yl)amino)-4-((2-methoxy-3-(1-methyl-1H-1,2,4-triazol-3-yl)phenyl)amino)-N-methylpyridazine-3-carboxamide (SHR2915)

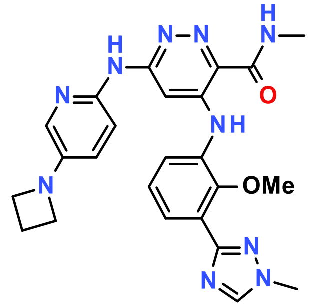

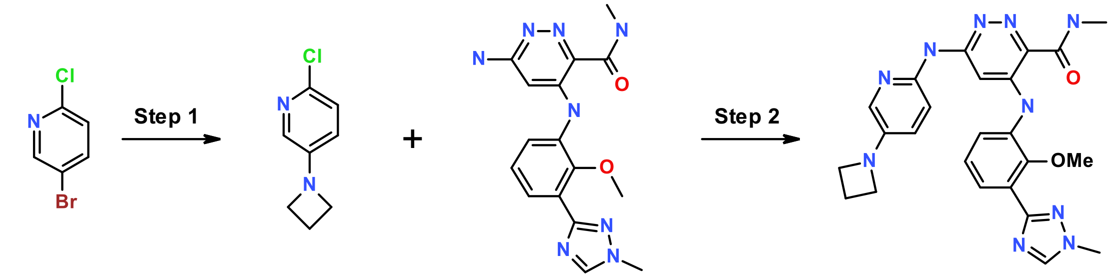

2A 2B 2C

Step 1

5-(azetidin-1-yl)-2-chloropyridine **2B**

A mixture of **2A** (500 mg, 2.6mmol, Bidepharm), azetidine (149 mg, 2.6 mmol, Bidepharm), $Pd_2(dba)_3$ (48 mg, 0.052 mmol, Greenchem), xantphos (90 mg, 0.156 mmol, Labnetwork) and t-BuONa (374 mg, 3.9 mmol, Aladdin) in toluene (12 mL, Richjoint) was evacuated and refilled with N_2 for 3 times. The resulting mixture was stirred at 100°C for 16h. The reaction mixture was direactly filtrated to remove the solid. Then the filtrate was cooled to room temperature, the resulting mixture was filtered to give title compound (316 mg, yield 72%) as a dark green solid.

LCMS: MS m/z (ESI): 169 $[M+H]^+$

Step 2

6-((5-(azetidin-1-yl)pyridin-2-yl)amino)-4-((2-methoxy-3-(1-methyl-1H-1,2,4-triazol-3-yl)phenyl)amino)-N-methylpyridazine-3-carboxamide

A mixture of **2B** (60 mg, 0.34 mmol, 8104-121), **2C** (60 mg, 0.17 mmol, Bidepharm), $Pd_2(dba)_3$ (31 mg, 0.034 mmol, Greenchem), xantphos (30 mg, 0.051mmol, Labnetwork) and Cs_2CO_3 (110 mg, 0.34 mmol, Aladdin) in dioxane (3 mL, Richjoint) was protected with N_2 and sealed. The resulting mixture was stirred at 135°C for 16h. The reaction was direactly filtrated to remove the solid. Then the filtrate was cooled to room temperature, the resulting mixture was filtered to give title compound (30 mg, yield 36%) as a yellow solid.

^{1}H NMR (400 MHz, DMSO-d_6): δ 10.95 (s, 1H), 9.80 (s, 1H), 9.08 (d, *J* = 4.8 Hz, 1H) 8.57 (s, 1H) 8.27 (s, 1H), 7.95 (d, *J* = 2.4 Hz, 1H), 7.63-7.59 (m, 2H), 7.51 (d, *J* = 8.8 Hz, 1H) ,7.45 (d, *J* = 2.8 Hz, 1H), 7.32 (t, *J* = 7.6 Hz, 1H), 6.92 (dd, *J* = 8.8 Hz, 2.8 Hz, 1H), 3.95 (s, 3H), 3.79 (t, *J* = 7.2 Hz, 4H), 3.75 (s, 3H), 2.85 (d, *J* = 4.8 Hz, 3H), 2.38-2.29 (m, 2H).

HPLC: 93.086% @ 254 nm, 89.457% @ 214 nm

LCMS: MS m/z (ESI): 487.2 $[M+H]^+$

6-(cyclopropanecarboxamido)-4-((3-(5-(((dimethyl(oxo)-l6-sulfaneylidene)amino)methyl)-1,2,4-oxadiazol-3-yl)-2-methoxyphenyl)amino)-N-(methyl-d3)pyridazine-3-carboxamide (SHR3290)

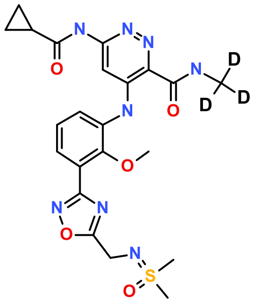

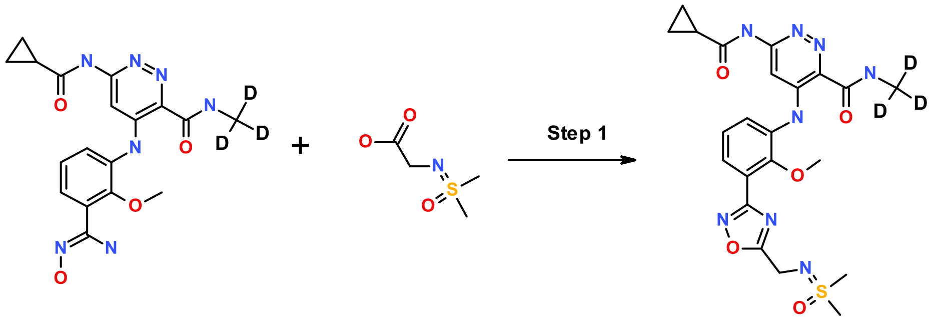

3A 3B

A mixture of (Z)-6-(cyclopropanecarboxamido)-4-((3-(N'-hydroxycarbamimidoyl)-2-methoxyphenyl)amino)-N-(methyl-d3)pyridazine-3-carboxamide **3A**[1] (250 mg，0.62 mol), 2-((dimethyl(oxo)-l6-sulfaneylidene)amino)acetic acid **3B**[2] (187 mg，1.2 mmol) and DIPEA (195 mg，1.55 mmol, Adamas) in DMF (5 mL) was stirred at room temperature for 90 min. Then the reaction was heated to 80°C for 4 hour. After cooling to room temperature and concentration under reduced pressure, the crude was purified by pre-HPLC to afford title compound (20mg, 0.039 mmol, yield 6.2%) as while solid.

LCMS: MS m/z (ESI): 518.1 [M+1]

^{1}H NMR (500 MHz, MeOD) δ 8.22 (s, 1H), 7.80 (d, 1H), 7.70 (d, 1H), 7.36 (t, 1H), 4.60 (s, 2H), 3.82 (s, 3H), 3.21 (s, 6H), 2.00-1.86 (m, 1H), 0.99-0.88 (m, 4H).

6-(cyclopropanecarboxamido)-4-((2-methoxy-3-(5-(S-methylsulfonimidoyl)-5,6-dihydro-4H-pyrrolo[3,4-d]thiazol-2-yl)phenyl)amino)-N-(methyl-d3)pyridazine-3-carboxamide (SHR3110)

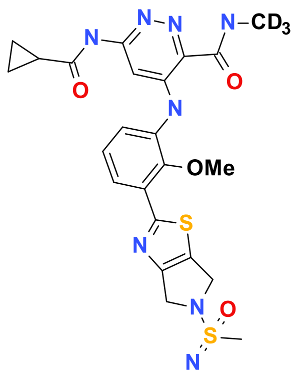

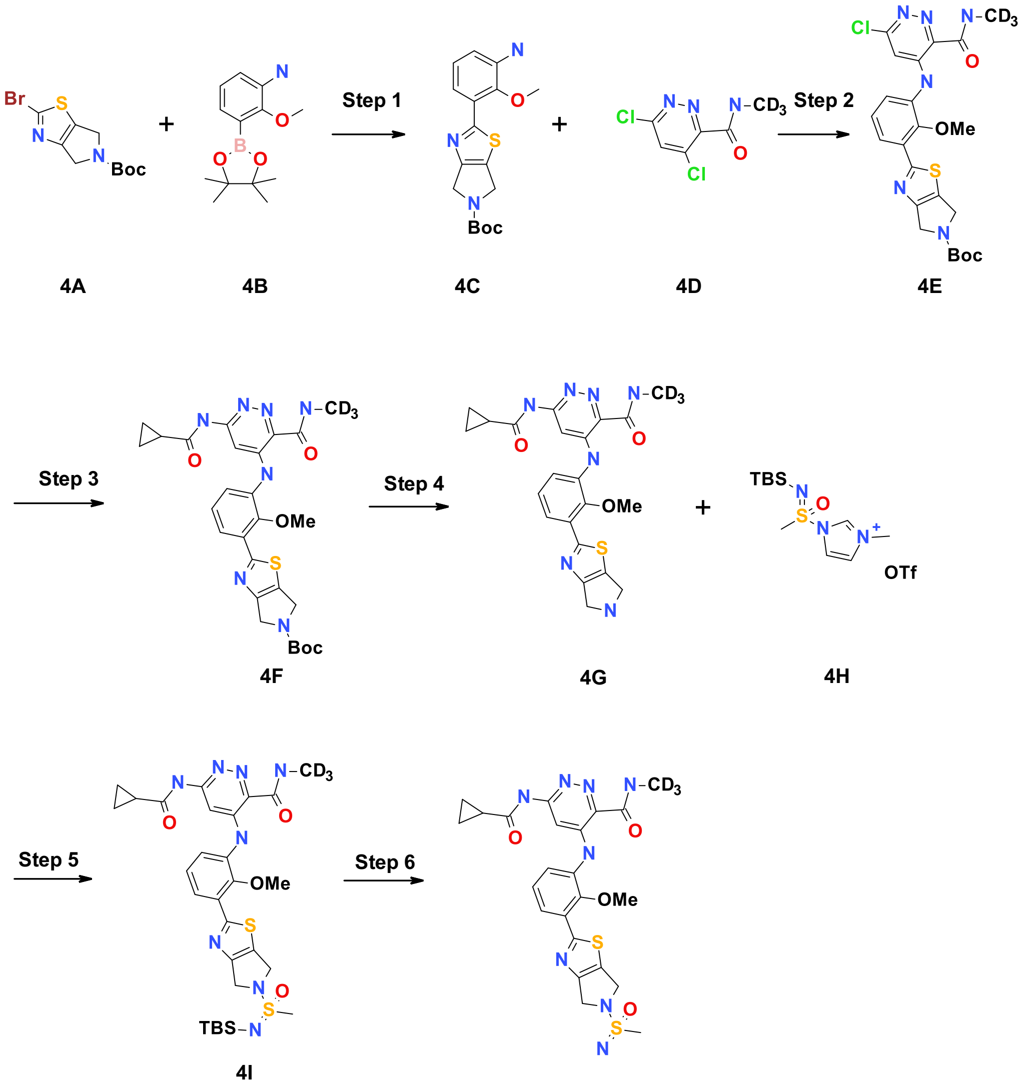

Step 1

tert-butyl 2-(3-amino-2-methoxyphenyl)-4,6-dihydro-5H-pyrrolo[3,4-d]thiazole-5-carboxylate **4C**

A mixture of tert-butyl 2-bromo-4,6-dihydro-5H-pyrrolo[3,4-d]thiazole-5-carboxylate **4A** (500 mg，1.64 mmol, Leyan), 2-methoxy-3-(4,4,5,5-tetramethyl-1,3,2-dioxaborolan-2-yl)aniline **4B** (612 mg，2.45mmol, Leyan), Pd(dppf)Cl_2 (121 mg，0.16 mmol, Shanghai Shaoyuan) and potassium carbonate (678 mg，4.91 mmol) in dioxane (10 mL) and water (1 mL) was stirred at 100°C under N_2 atmosphere for 2 hours. After concentration, the crude was purified by silica gel chromatography to afford title compound (500 mg, yield 87%) as white solid.

LCMS: MS m/z (ESI): 348.0 $[M+1]^+$

Step 2

tert-butyl 2-(3-((6-chloro-3-((methyl-d3)carbamoyl)pyridazin-4-yl)amino)-2-methoxyphenyl)-4,6-dihydro-5H-pyrrolo[3,4-d]thiazole-5-carboxylate **4E**

To a solution of **4C** (250 mg，0.72 mmol), **4D** [3] (150 mg，0.72 mmol) in THF (5 mL) was added LHMDS (1.79 mL，1.79 mmol，1.0 M in THF, Adamas) dropwise and the reaction was stirred at room temperature for 1 hour. Saturated NH4Cl solution and EtOAc (30 mL×3) was added. The organic layer was collected, washed with brine and dried over Na2SO4. After concentration under reduced pressure, the crude was purified by silica gel chromatography to afford title compound (250 mg, yield 67%) as white solid.

LCMS: MS m/z (ESI): 520.0 $[M+1]^+$

Step 3

tert-butyl 2-(3-((6-(cyclopropanecarboxamido)-3-((methyl-d3)carbamoyl)pyridazin-4-yl)amino)-2-methoxyphenyl)-4,6-dihydro-5H-pyrrolo[3,4-d]thiazole-5-carboxylate **4F**

A mixture of **4E** (250 mg，0.48 mmol), cyclopropanecarboxamide (204 mg，2.39 mmol，Shanghai Shaoyuan), $Pd_2(dba)_3$ (44 mg，0.046 mmol, Shanghai Shaoyuan), Xantphos (24 mg，0.046 mmol, Shanghai Shaoyuan) and cesium carbonate (626 mg，1.92 mmol, Shanghai Shaoyuan) in dioxane (5mL) was stirred at 110°C microwave condition under N_2 atmosphere for 1hour. After concentration under reduced pressure, the crude was purified by silica gel chromatography to afford title compound (90 mg, yield 33%) as yellow solid.

LCMS: MS m/z (ESI): 569.1 $[M+1]^+$

Step 4

6-(cyclopropanecarboxamido)-4-((3-(5,6-dihydro-4H-pyrrolo[3,4-d]thiazol-2-yl)-2-methoxyphenyl)amino)-N-(methyl-d3)pyridazine-3-carboxamide **4G**

A mixture of **4F** (80 mg，0.14 mmol) and HCl (16 mmol, 4 mL, 4M in dioxane, Adamas) was stirred at room temperature for 1 hour. After concentration, the crude tile compound was obtained and used directly to next step without further purification.

LCMS: MS m/z (ESI): 469.1 $[M+1]^+$

Step 5

4-((3-(5-(N-(tert-butyldimethylsilyl)-S-methylsulfonimidoyl)-5,6-dihydro-4H-pyrrolo[3,4-d]thiazol-2-yl)-2-methoxyphenyl)amino)-6-(cyclopropanecarboxamido)-N-(methyl-d3)pyridazine-3-carboxamide **4I**

A mixture of **4G** (72 mg，0.15 mmol), 1-(N-(tert-butyldimethylsilyl)-S-methylsulfonimidoyl)-3-methyl-1H-imidazol-3-ium trifluoromethanesulfonate **4H** [4] (590 mg，1.39 mmol) and Et_3N (78 mg，0.77 mmol) in DMF was stirred at 40°C overnight. After concentration, the crude was purified by silica gel chromatography to afford title compound 6I (30 mg, yield 30%) as yellow solid.

LCMS: MS m/z (ESI): 660.1 $[M+1]^+$

Step 6

6-(cyclopropanecarboxamido)-4-((2-methoxy-3-(5-(S-methylsulfonimidoyl)-5,6-dihydro-4H-pyrrolo[3,4-d]thiazol-2-yl)phenyl)amino)-N-(methyl-d3)pyridazine-3-carboxamide

A mixture of **4I** (30 mg，0.045 mmol) and HCl (8 mmol, 2 mL, 4M in dioxane, Adamas) was stirred at room temprature for 1 hour. After concentration, the crude was purified by pre-HPLC to afford title compound (5 mg, yield 20%) as yellow solid.

LCMS: MS m/z (ESI): 546.0 $[M+1]^+$

^{1}H NMR (500 MHz, DMSO-d_6) δ 11.35 (s, 1H), 10.97 (s, 1H), 9.19 (s, 1H), 8.10 (s, 1H), 8.03-8.04 (m, 1H), 7.54-7.56 (m, 1H), 7.32-7.34 (m, 1H), 4.54-4.68 (m, 4H), 3.78 (s, 3H), 2.91 (s, 3H) , 1.24 (m, 1H), 0.79-0.83 (m, 4H)

Reference:

1. Spergel HS, Pitts JW, Mertzman, EM, Moslin MR, Sherwood CT, Gilmore LJ, Dyckman, JA. Amide-substituted herterocyclic compounds for the treatment of conditions related to the modulation of IL-12, IL-23 and/or IFN-alha. WO2020092196 A1. Page 60.
2. Goldberg WF, Kettle GJ, Xiong J, Lin D. General synthetic strategies towards N-alkyl sulfoximine building blocks for medicinal chemistry and the use of dimethylsulfoximine as a versatile precursor. *Tetrahedron* (2014) *70:* 6613-6622. Epub 2014/09/16. .Doi: 10.1016/j.tet.2014.06.120.
3. Liu C, Yang GM, Xiao Z, Chen L, Moslin MR, Tokarski, SJ, Weinstein SD. Sulfone pyridine alkyl amides-substituted herteroaryl compounds. US20190152948A1. Paragraph 0426-0434.
4. Zasukha SV, Timoshenko VM, Tolmachev AA, Pivnytska VO, Gavrylenko O, Zhersh S, Shermolovich Y, Grygorenko OO. Sulfonimidamides and Imidosulfuric Diamides: Compounds from an Underexplored Part of Biologically Relevant Chemical Space. *Chemistry* (2019) *25*(28):6928-6940. Epub 2019/04/01. Doi: 10.1002/chem.201900440.

Supplementary Figure 1. TYK2/JAK-mediated cytokine signaling pathways and therapeutic TYK2/JAK inhibitors

Type I and II cytokine receptors physically associate with JAKs, which can transduce the extracellular ligand signals into the cell. Different receptors associate with different JAK family members, so that selective blockade of one JAK can inhibit a specific biologic function while leaving the other JAK-dependent cytokine signal unaffected, whereas nonselective JAK inhibitors inhibit multiple cytokines simultaneously.

Abbreviations: DC, Dendritic cell; EPO, erythropoietin; GH, growth hormone; GM-CSF, granulocyte-macrophage colony-stimulating factor; IFN, interferon; IFN-γ, interferon gamma; IL, interleukin; JAK, Janus kinase; MHC: Major histocompatibility complex; Th, T helper cell; TYK, tyrosine kinase; TPO, thrombopoietin; Treg: Regulatory T cell.

Supplementary Figure 2. Activities of TYK2 JH2 ligands to suppress IFNα-induced-TYK2-associated signaling pathway

Representative dose-response curves of TYK2 JH2 ligands, SHR8551, 2396 and 9332 in blocking IFNα-induced-IRF activities in Jurkat reporter cells. Jurkat-Dual™ T cells with a stably integrated IRF-dependent secreted embryonic alkaline phosphatase (SEAP) reporter gene were treated with serially diluted compounds and stimulated with human recombinant IFNα overnight. Activity of SEAP was then detected from the cell cultured supernatant for the measurement of IFNα-mediated-IRF activities. Inhibition data were calculated by comparison to vehicle control wells for 0% inhibition and non-stimulated controls for 100% inhibition. Dose response curves were

generated by non-linear regression analysis to determine the IC_{50} values of compounds using GraphPad Prism.

Supplementary Figure 3. TYK2 JH2 compounds showed broad cross-species activities (Monkey, Canine, Rat and Mouse)

(A) Suppression of IL23-induced-IL17 production in mouse splenocytes by TYK2 JH2 ligands, SHR2915, 0936, 1039 and 1213; (B-E) Suppression of IL12-induced-IFNγ production by TYK2 JH2 ligands, SHR2915, 0936, 1039 and 1213, in mouse splenocytes (B), Canine PBMCs (C), Rat PBMCs (D) and Macaque PBMCs (E). Inhibition data were calculated by comparison to vehicle control wells for 0% inhibition and non-stimulated control wells for 100% inhibition. Dose response curves were then generated to determine the IC_{50} values as derived by non-linear regression analysis using GraphPad Prism.

Supplementary Figure 4. TYK2 JH1/JH2 binding for Tofacitinib

Representative dose-response curves for binding affinity of Tofacitinib (CP03) to TYK2 JH1 kinase domain (A and B) and JH2 pseudokinase domain (C and D) assessed by KdELECT competition binding assay (DiscoverX). The figures are representative of two independent replicates within this experiment. Dissociation constants (Kds) for test compound-JH1 or -JH2 domain interactions are calculated by measuring the amount of isolated JH1 or JH2 protein segments captured on the solid support as function of the test compound concentrations.

Supplementary Figure 5. JAK1/2/3, TYK2 JH1/JH2 binding for BMS165 (Deucravacitinib), PF841 (Brepocitinib), PF647 (Ropsacitinib), SHR0936, 2915, 1039, and 1213

Representative dose-response curves for binding affinity of BMS165, PF841, PF647, SHR0986, 2915, 1039 and 1213 to JAK1,JAK2,JAK3 and TYK2 JH1 kinase domain and JH2 pseudokinase domain assessed by KdELECT competition binding assay (DiscoverX). The figures are representative of two independent replicates within this experiment. Dissociation constants (Kds) for test compound-JH1 or -JH2 domain interactions are calculated by measuring the amount of isolated JH1 or JH2 protein segments captured on the solid support as function of the test compound concentrations.

Supplementary Figure 6. Inhibition of IFNα- or IL23- induced TYK2-associated TYK2-mediated cellular activities by JAK/TYK2 inhibitors, SHR3110, Filgotinib, Baricitinib, Upadacitinib, Ruxolitinib, and Deucravacitinib

Suppression of IFNα-induced-STAT1 (Tyr701) phosphorylation (A) and IL23-induced STAT3 (Tyr705) phosphorylation (B) by SHR3110, Filgotinib, Baricitinib, Upadacitinib, Ruxolitinib, and Deucravacitinib in Kit225 cells. Inhibition data were calculated by comparison to vehicle control wells for 0% inhibition and non-stimulated control wells for 100% inhibition. Dose response curves were then generated to determine the IC_{50} values as derived by non-linear regression analysis using GraphPad Prism.

Supplementary Figure 7. Suppression of IFNα-induced TYK2-associated functional activities in human whole blood by TYK2/JAK inhibitors, SHR3110, Filgotinib, Baricitinib, Upadacitinib, Ruxolitinib, and Deucravacitinib

Suppression of IFNα-stimulated-CXCL10 production in human whole blood samples from three different donors (A, B, and C). Human whole blood samples were treated with serially diluted compounds and stimulated by recombinant human IFNα for 16 hours. The plasma was collected for detection of CXCL10 production by AlphaLISA assay. Inhibition data were calculated by comparison to vehicle control wells for 0% inhibition and non-stimulated control wells for 100% inhibition. Dose response curves were generated non-linear regression analysis using GraphPad Prism to determine the IC_{50} value of each compound.

Supplementary Table 1. KINOMEscanEdge profiling for SHR1274

Binding affinity of SHR1274 to each kinase or pseudokinase was assessed using the DiscoverX scanEdge screening. The compound was tested at a concentration of 1,000 nM in competition binding assays at Eurofins DiscoverX Corporation following the established standard protocol (DiscoverX, San Diego, CA). This profiles compound selectivity against a panel of 98 kinases including kinases from the AGC, CAMK, CMGC, CK1, STE, TK, TKL, lipid, and atypical kinase families, and other mutant forms.

Supplementary Table 2. DMPK profile of SHR2396, 8751, 9332, 2178, 4995, and 4997

Supplementary Figure 1.

TYK2/JAK-mediated cytokine signaling pathways and therapeutic TYK2/JAK inhibitors

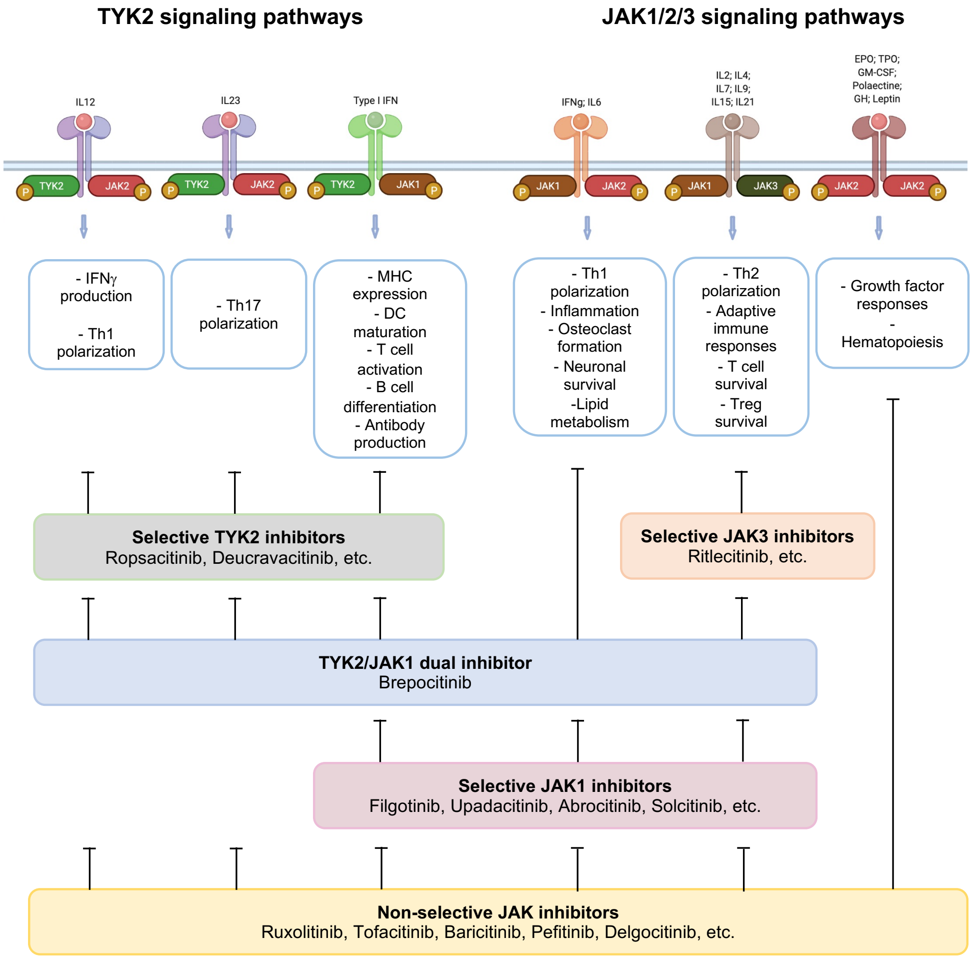

Supplementary Figure 2.

Activities of TYK2 JH2 ligands to suppress IFNα-induced-TYK2-associated signaling pathway

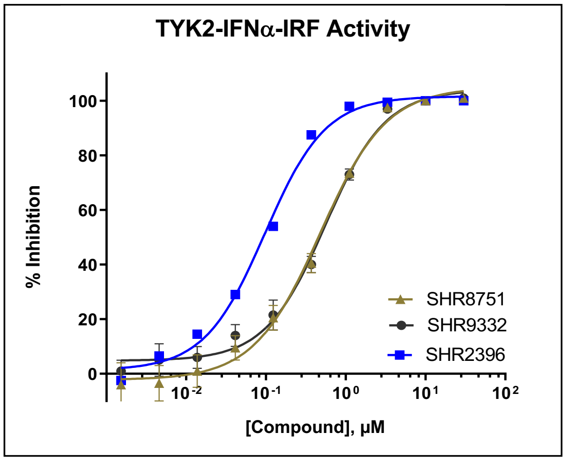

TYK2-associated IFNα-IRF Pathway Suppression in Jurkat Dual-Reporter Cells

Compd/ IC_{50}, nM	IFNα-IRF activity
SHR8751	48
SHR9332	55
SHR2396	9.7

Supplementary Figure 3.

TYK2 JH2 compounds showed broad cross-species activities (Macaque, Canine, Rat, and Mouse)

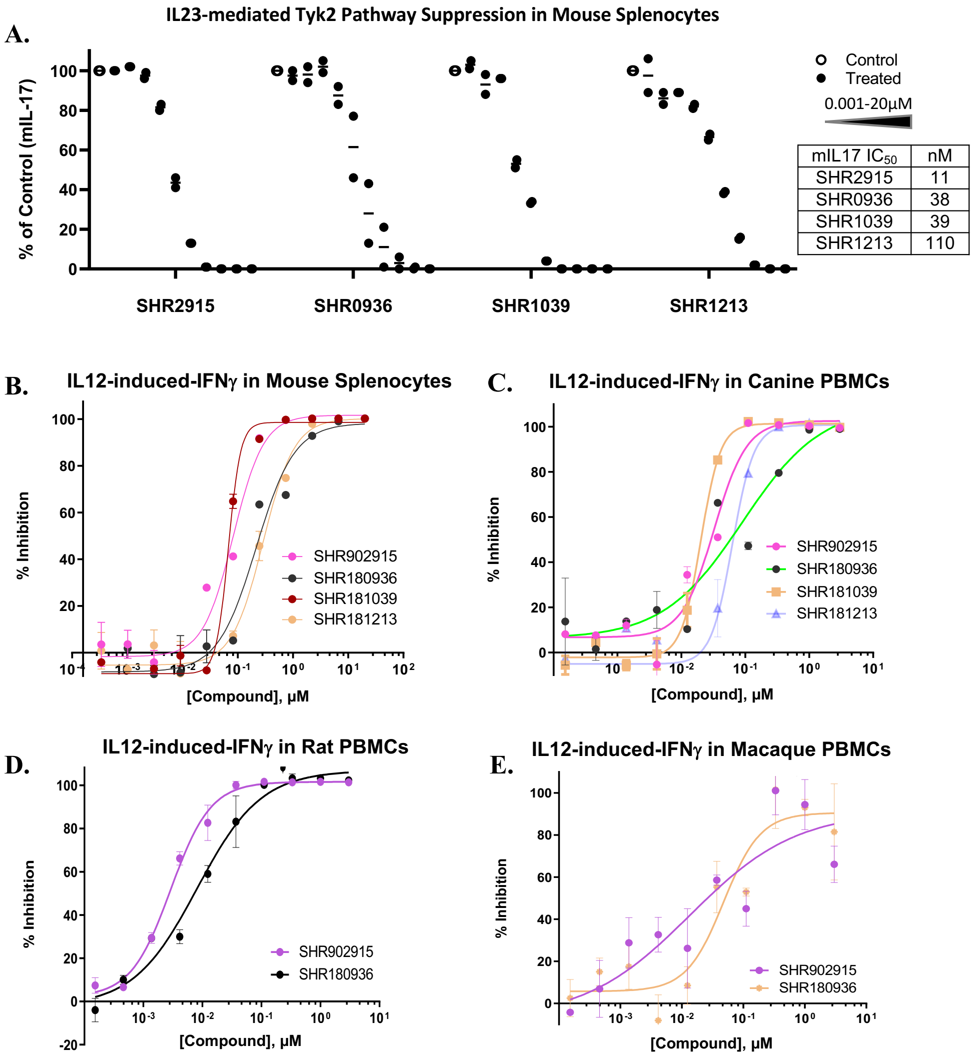

Cross-species Activity of TYK2 JH2 Ligands to Suppress TYK2-mediated IL12 Pathway

	IL12-induced-IFNγ, IC_{50}, nM			
**Compound/Species**	**Mouse**	**Canine**	**Rat**	**Monkey**
SHR2915	60	33	3	14
SHR0936	200	90	8	48
SHR1039	37	20		
SHR1213	190	63		

Supplementary Figure 4.

TYK2 JH1/JH2 binding by Tofacitinib

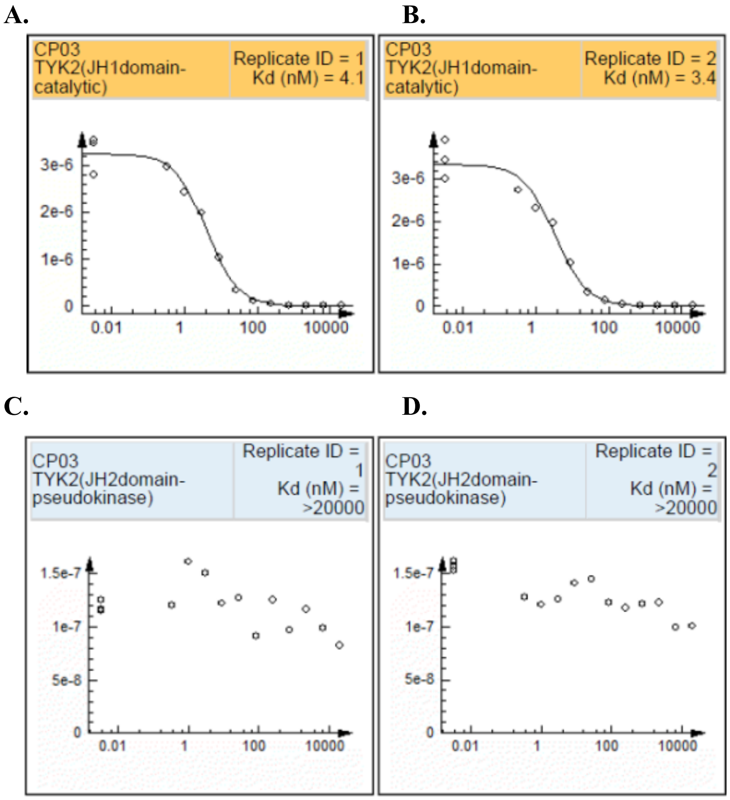

TYK2 Kinase (JH1) and Pseudokinase (JH2) Domain Binding by Tofacitinib* (Kd, nM)

JH1	JH2
3.7	>20000

* CP03

Supplementary Figure 5.

JAK1/2/3, TYK2 JH1/JH2 binding for BMS165 (Deucravacitinib), PF841 (Brepocitinib), PF647 (Ropsacitinib), SHR0936, 2915, 1039, and 1213

Compound	JAK1(JH1domain-catalytic)	JAK1(JH2domain-pseudokinase)	JAK2(JH1domain-catalytic)	JAK2(JH2domain-pseudokinase)	JAK3(JH1domain-catalytic)	TYK2(JH1domain-catalytic)	TYK2(JH2domain-pseudokinase)
Compound Name	Kd (nM)	Kd (nM)	Kd (nM)	Kd (nM)	Kd (nM)	Kd (nM)	Kd (nM)
Brepocitinib	3.1	4800	0.38	>20000	18	0.13	1600
SHR180936-000-00	>20000	1.1	>20000	860	4600	>20000	0.0093
SHR181039-000-53	>20000	0.23	650	76	390	19000	0.0063
SHR181213-000-18	>20000	12	>20000	8300	>20000	>20000	0.0054
Deucravacitinib	>20000	0.2	1100	15	500	13000	0.0038
SHR902915-000-53	>20000	0.35	980	160	450	13000	0.017
Ropsacitinib	48	460	0.26	7000	30	0.047	100

Kd Legend

x<100nM	100nM≤x<1uM	x≥1uM	No Binding	Not Requested

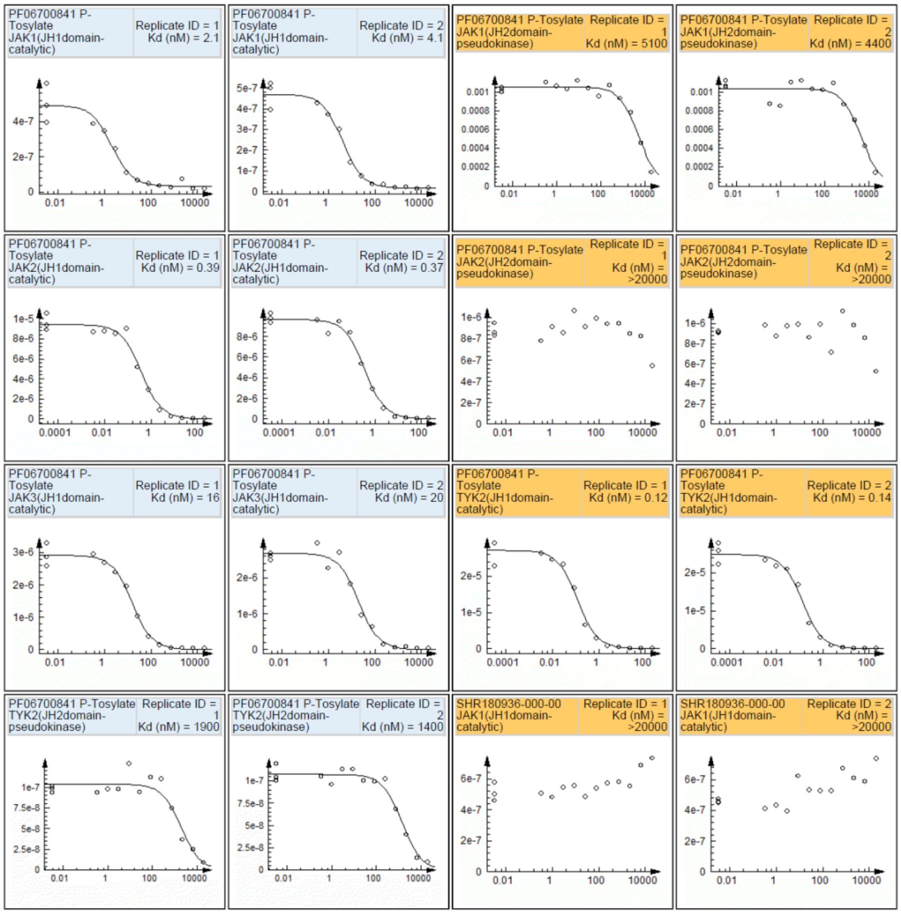

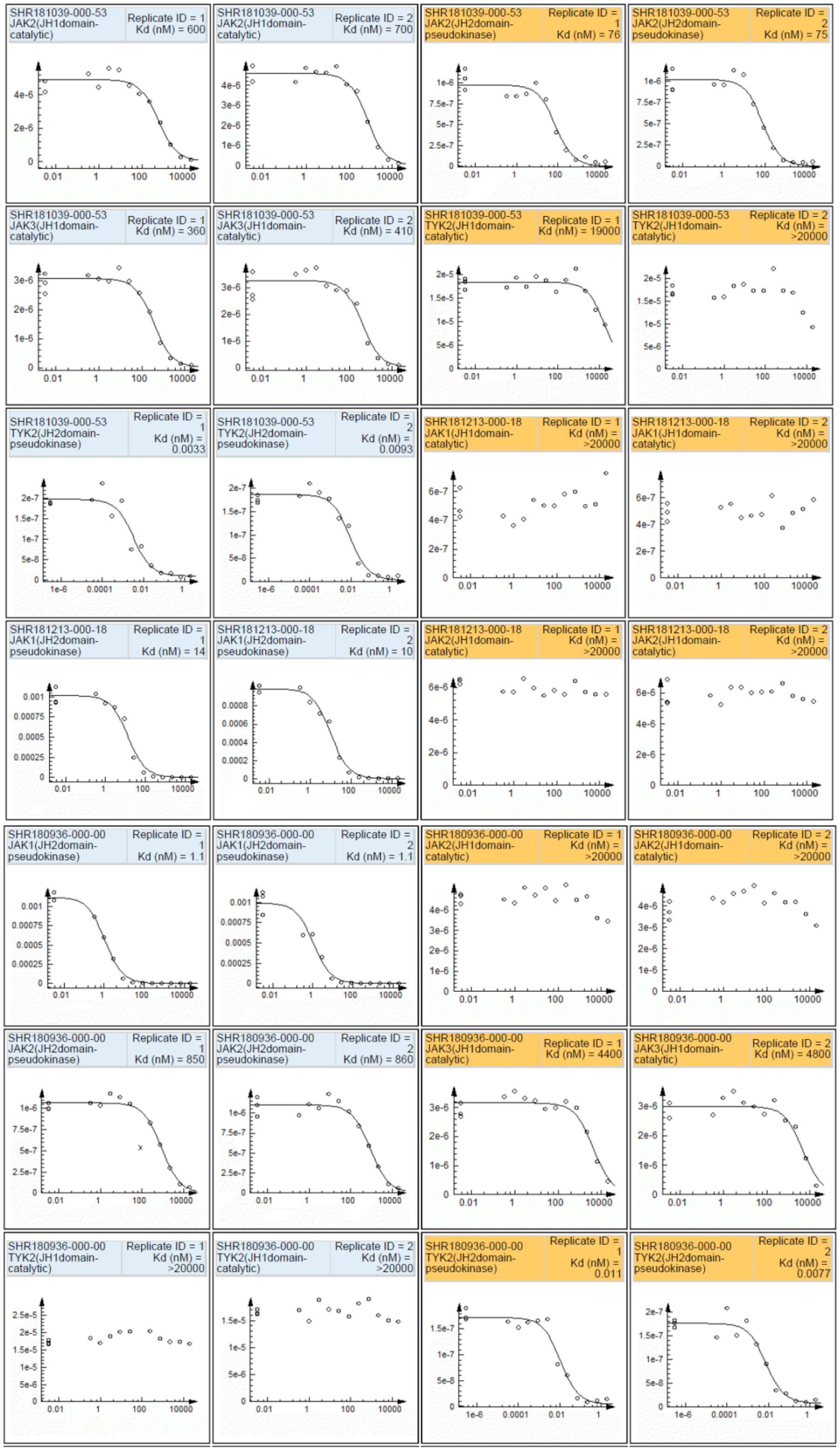
SHR181039-000-53 JAK2(JH1domain-catalytic) Replicate ID = 1 Kd (nM) = 600
SHR181039-000-53 JAK2(JH1domain-catalytic) Replicate ID = 2 Kd (nM) = 700
SHR181039-000-53 JAK2(JH2domain-pseudokinase) Replicate ID = 1 Kd (nM) = 76
SHR181039-000-53 JAK2(JH2domain-pseudokinase) Replicate ID = 2 Kd (nM) = 75
SHR181039-000-53 JAK3(JH1domain-catalytic) Replicate ID = 1 Kd (nM) = 360
SHR181039-000-53 JAK3(JH1domain-catalytic) Replicate ID = 2 Kd (nM) = 410
SHR181039-000-53 TYK2(JH1domain-catalytic) Replicate ID = 1 Kd (nM) = 19000
SHR181039-000-53 TYK2(JH1domain-catalytic) Replicate ID = 2 Kd (nM) = >20000
SHR181039-000-53 TYK2(JH2domain-pseudokinase) Replicate ID = 1 Kd (nM) = 0.0033
SHR181039-000-53 TYK2(JH2domain-pseudokinase) Replicate ID = 2 Kd (nM) = 0.0093
SHR181213-000-18 JAK1(JH1domain-catalytic) Replicate ID = 1 Kd (nM) = >20000
SHR181213-000-18 JAK1(JH1domain-catalytic) Replicate ID = 2 Kd (nM) = >20000
SHR181213-000-18 JAK1(JH2domain-pseudokinase) Replicate ID = 1 Kd (nM) = 14
SHR181213-000-18 JAK1(JH2domain-pseudokinase) Replicate ID = 2 Kd (nM) = 10
SHR181213-000-18 JAK2(JH1domain-catalytic) Replicate ID = 1 Kd (nM) = >20000
SHR181213-000-18 JAK2(JH1domain-catalytic) Replicate ID = 2 Kd (nM) = >20000
SHR180936-000-00 JAK1(JH2domain-pseudokinase) Replicate ID = 1 Kd (nM) = 1.1
SHR180936-000-00 JAK1(JH2domain-pseudokinase) Replicate ID = 2 Kd (nM) = 1.1
SHR180936-000-00 JAK2(JH1domain-catalytic) Replicate ID = 1 Kd (nM) = >20000
SHR180936-000-00 JAK2(JH1domain-catalytic) Replicate ID = 2 Kd (nM) = >20000
SHR180936-000-00 JAK2(JH2domain-pseudokinase) Replicate ID = 1 Kd (nM) = 850
SHR180936-000-00 JAK2(JH2domain-pseudokinase) Replicate ID = 2 Kd (nM) = 860
SHR180936-000-00 JAK3(JH1domain-catalytic) Replicate ID = 1 Kd (nM) = 4400
SHR180936-000-00 JAK3(JH1domain-catalytic) Replicate ID = 2 Kd (nM) = 4800
SHR180936-000-00 TYK2(JH1domain-catalytic) Replicate ID = 1 Kd (nM) = >20000
SHR180936-000-00 TYK2(JH1domain-catalytic) Replicate ID = 2 Kd (nM) = >20000
SHR180936-000-00 TYK2(JH2domain-pseudokinase) Replicate ID = 1 Kd (nM) = 0.011
SHR180936-000-00 TYK2(JH2domain-pseudokinase) Replicate ID = 2 Kd (nM) = 0.0077

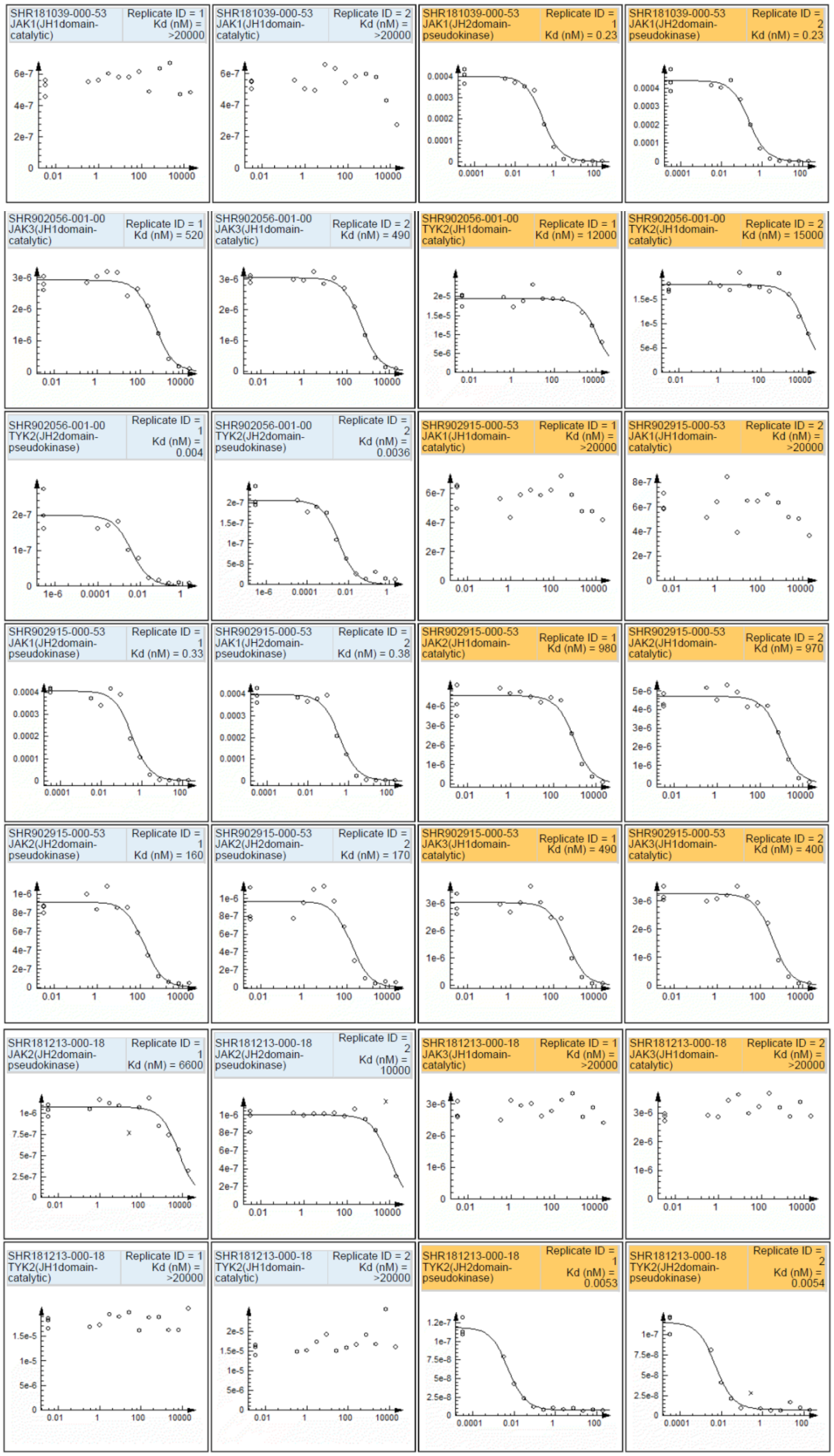
SHR181039-000-53 JAK1(JH1domain-catalytic) Replicate ID = 1 Kd (nM) = >20000
SHR181039-000-53 JAK1(JH1domain-catalytic) Replicate ID = 2 Kd (nM) = >20000
SHR181039-000-53 JAK1(JH2domain-pseudokinase) Replicate ID = 1 Kd (nM) = 0.23
SHR181039-000-53 JAK1(JH2domain-pseudokinase) Replicate ID = 2 Kd (nM) = 0.23
SHR902056-001-00 JAK3(JH1domain-catalytic) Replicate ID = 1 Kd (nM) = 520
SHR902056-001-00 JAK3(JH1domain-catalytic) Replicate ID = 2 Kd (nM) = 490
SHR902056-001-00 TYK2(JH1domain-catalytic) Replicate ID = 1 Kd (nM) = 12000
SHR902056-001-00 TYK2(JH1domain-catalytic) Replicate ID = 2 Kd (nM) = 15000
SHR902056-001-00 TYK2(JH2domain-pseudokinase) Replicate ID = 1 Kd (nM) = 0.004
SHR902056-001-00 TYK2(JH2domain-pseudokinase) Replicate ID = 2 Kd (nM) = 0.0036
SHR902915-000-53 JAK1(JH1domain-catalytic) Replicate ID = 1 Kd (nM) = >20000
SHR902915-000-53 JAK1(JH1domain-catalytic) Replicate ID = 2 Kd (nM) = >20000
SHR902915-000-53 JAK1(JH2domain-pseudokinase) Replicate ID = 1 Kd (nM) = 0.33
SHR902915-000-53 JAK1(JH2domain-pseudokinase) Replicate ID = 2 Kd (nM) = 0.38
SHR902915-000-53 JAK2(JH1domain-catalytic) Replicate ID = 1 Kd (nM) = 980
SHR902915-000-53 JAK2(JH1domain-catalytic) Replicate ID = 2 Kd (nM) = 970
SHR902915-000-53 JAK2(JH2domain-pseudokinase) Replicate ID = 1 Kd (nM) = 160
SHR902915-000-53 JAK2(JH2domain-pseudokinase) Replicate ID = 2 Kd (nM) = 170
SHR902915-000-53 JAK3(JH1domain-catalytic) Replicate ID = 1 Kd (nM) = 490
SHR902915-000-53 JAK3(JH1domain-catalytic) Replicate ID = 2 Kd (nM) = 400
SHR181213-000-18 JAK2(JH2domain-pseudokinase) Replicate ID = 1 Kd (nM) = 6600
SHR181213-000-18 JAK2(JH2domain-pseudokinase) Replicate ID = 2 Kd (nM) = 10000
SHR181213-000-18 JAK3(JH1domain-catalytic) Replicate ID = 1 Kd (nM) = >20000
SHR181213-000-18 JAK3(JH1domain-catalytic) Replicate ID = 2 Kd (nM) = >20000
SHR181213-000-18 TYK2(JH1domain-catalytic) Replicate ID = 1 Kd (nM) = >20000
SHR181213-000-18 TYK2(JH1domain-catalytic) Replicate ID = 2 Kd (nM) = >20000
SHR181213-000-18 TYK2(JH2domain-pseudokinase) Replicate ID = 1 Kd (nM) = 0.0053
SHR181213-000-18 TYK2(JH2domain-pseudokinase) Replicate ID = 2 Kd (nM) = 0.0054

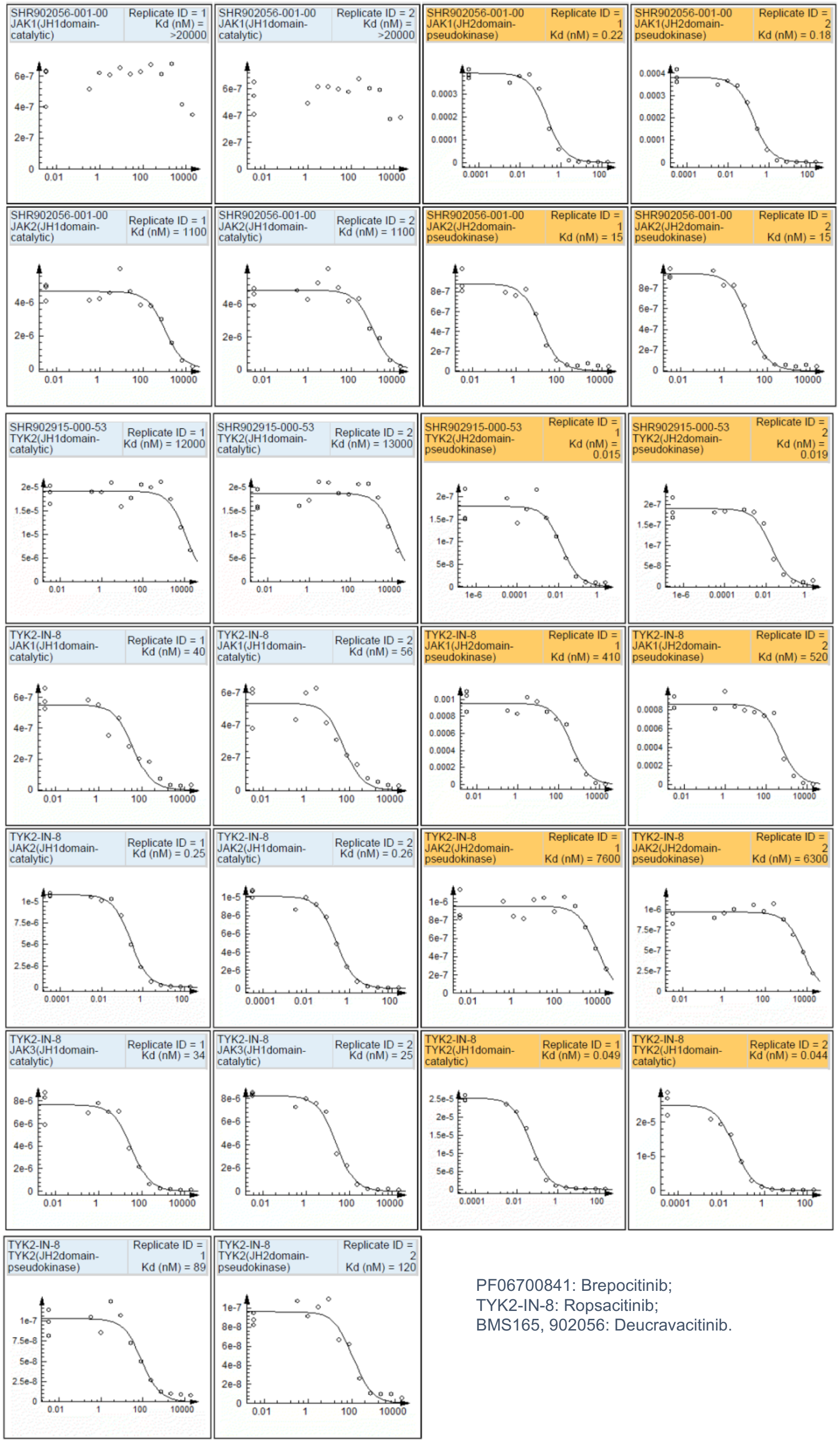

PF06700841: Brepocitinib;
TYK2-IN-8: Ropsacitinib;
BMS165, 902056: Deucravacitinib.

Supplementary Figure 6.

Inhibition of IFNα- or IL23- induced TYK2-associated TYK2-mediated cellular activities by JAK/TYK2 inhibitors, SHR3110, Filgotinib, Baricitinib, Upadacitinib, Ruxolitinib, and Deucravacitinib

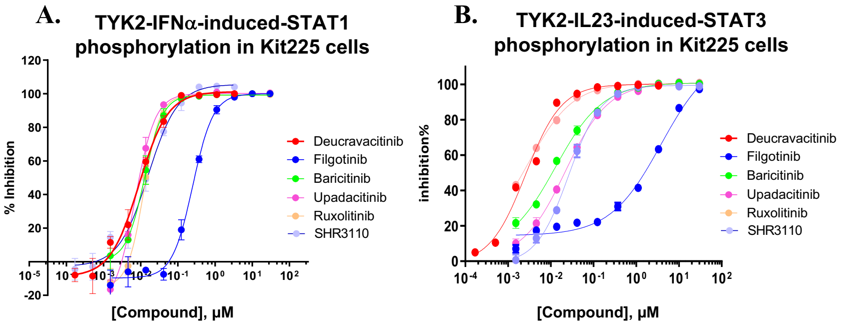

TYK2-associated Cellular Cytokine Activity Suppression

Compd/ IC_{50}, nM	IFNα-induced-STAT1 phosphorylation	IL23-induced-STAT3 phosphorylation
SHR3110	16	24
Deucravacitinib	8.5	5.0
Filgotinib	250	3800
Baricitinib	13	13
Upadacitinib	7	22
Ruxolitinib	12	3.0

Supplementary Figure 7.

Suppression of IFNα-induced TYK2-associated functional activities in human whole blood by TYK2/JAK inhibitors, SHR3110, Filgotinib, Baricitinib, Upadacitinib, Ruxolitinib, and Deucravacitinib

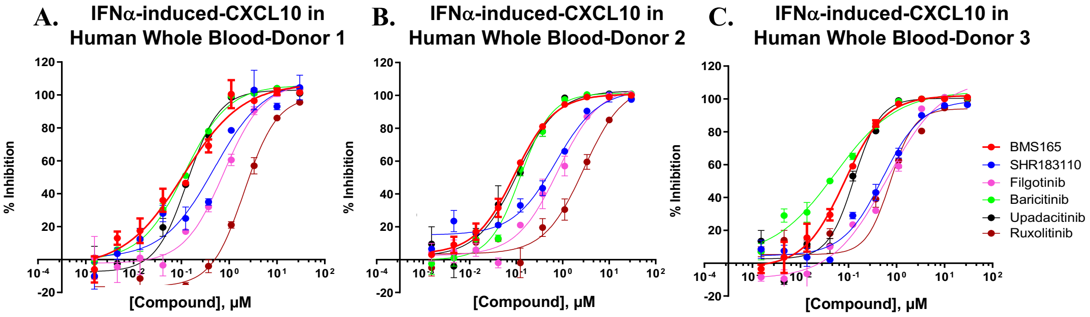

TYK2-associated IFNα-induced-CXCL10 Production in Human Whole Blood

Compounds/IC_{50}, nM	Donor1	Donor 2	Donor 3	Average
Deucravacitinib (BM165)	110	94	83	96
SHR183110	480	710	520	570
Filgotinib	770	790	630	730
Baricitinib	120	120	53	100
Upadacitinib	130	110	130	120
Ruxolitinib	2100	2800	740	1900

Supplementary Table 1.

KINOMEscanEdge profiling for SHR1274

Target	SHR181274-000-00
Gene Symbol	%Ctrl @ 1000nM
ABL1(E255K)-phosphorylated	80
ABL1(T315I)-phosphorylated	94
ABL1-nonphosphorylated	69
ABL1-phosphorylated	83
ACVR1B	100
ADCK3	100
AKT1	100
AKT2	100
ALK	100
AURKA	100
AURKB	76
AXL	99
BMPR2	92
BRAF	100
BRAF(V600E)	77
BTK	88
CDK11	100
CDK2	100
CDK3	90
CDK7	100
CDK9	100
CHEK1	100
CSF1R	100
CSNK1D	100
CSNK1G2	100
DCAMKL1	96
DYRK1B	99
EGFR	100
EGFR(L858R)	100
EPHA2	100
ERBB2	100
ERBB4	100
ERK1	100
FAK	100
FGFR2	100
FGFR3	100
FLT3	95
GSK3B	78
IGF1R	100
IKK-alpha	100
IKK-beta	100
INSR	100
JAK2(JH1domain-catalytic)	98
JAK3(JH1domain-catalytic)	62
JNK1	79
JNK2	96
JNK3	73
KIT	100

Target	SHR181274-000-00
Gene Symbol	%Ctrl @ 1000nM
KIT(D816V)	100
KIT(V559D,T670I)	100
LKB1	100
MAP3K4	100
MAPKAPK2	100
MARK3	84
MEK1	83
MEK2	78
MET	100
MKNK1	93
MKNK2	98
MLK1	100
p38-alpha	100
p38-beta	100
PAK1	100
PAK2	87
PAK4	100
PCTK1	100
PDGFRA	86
PDGFRB	99
PDPK1	100
PIK3C2B	100
PIK3CA	100
PIK3CG	100
PIM1	100
PIM2	100
PIM3	100
PKAC-alpha	100
PLK1	100
PLK3	90
PLK4	100
PRKCE	98
RAF1	100
RET	100
RIOK2	85
ROCK2	100
RSK2(Kin.Dom.1-N-terminal)	30
SNARK	90
SRC	100
SRPK3	86
TGFBR1	100
TIE2	100
TRKA	64
TSSK1B	100
TYK2(JH1domain-catalytic)	100
ULK2	89
VEGFR2	72
YANK3	77
ZAP70	97

Supplementary Table 2.

DMPK profile of SHR2396, 8751, 9332 2178, 4995, and 4997

ID	SHR2396	SHR8751	SHR9332	SHR2178
H/R/M/D/Mk LM $T_{1/2}$ (min)	>139/>67/31/ >139/50	>139/>67/>139/ >139/>99	47.5/>67/49.8 /46.5/30.9	34/24/55/40/99
H/R/M/D/Mk PPB (%unbound)	2.58/4.74/2.92/ 4.34/3.51	40.2/32.9/35/ 37.7/28.1	27.4/25.4/24.9/ 32.5/23.1	
Solubility (µM) (PBS/Fas/Fes)	0.34/8.6/8.2	17.6/60.7/60.9	25.6/71.4/108	0.1/1.7/3.5
LogD	1.19	0.43	0.48	
CYP450 (µM) 1A2/2C9/2D6/3A4(m)/3A4(t)/2C19	all >30	all >30	all >30	4/30/30/30/30/30
hERG IC_{50} (µM)	>30	>30	>30	
GSH	negative	negative	negative	negative

ID	SHR4995	SHR4997
MW / JClogP/ PSA/LogD	529.59/-1.49/183.8/-	529.59/-1.49/183.8/-
Tyk2 JH2 Binding Kd (nM)	0.0034	0.0043
IL-23_Kit225_pSTAT3 IC_{50} (nM)	39, 53	43, 49
IFNa_Kit225_pSTAT1 IC_{50} (nM)	32, 26	37, 28
IFNa_WB_CXCL10 IC_{50} (nM)	290	280
hERG IC_{50} (uM)	>30	19
Mouse PK @ 2mpk Cmax / AUC / T1/2/ F / CL	102.9/ 138.0/ 1.63/ 4.36%/ 10.5	74.9/ 117.9/ 1.99/ 4.92%/ 13.9
Mouse High Dose PK Cmax / AUC / T1/2	92.8/ 452/ 4.24 @ 15 mpk	328 871/ 3.92 @ 15 mpk

SHR4995

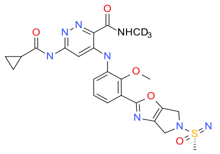

SHR4997

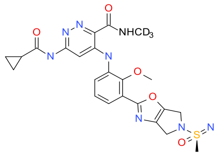

Supplement: Supplementary file 1 [file DataSheet_1.pdf]
